# TNF-α Induced by Hepatitis C Virus via TLR7 and TLR8 in Hepatocytes Supports Interferon Signaling via an Autocrine Mechanism

**DOI:** 10.1371/journal.ppat.1004937

**Published:** 2015-05-29

**Authors:** Jiyoung Lee, Yongjun Tian, Stephanie Tze Chan, Ja Yeon Kim, Cecilia Cho, Jing-hsiung James Ou

**Affiliations:** Department of Molecular Microbiology and Immunology, University of Southern California, Keck School of Medicine, Los Angeles, California, United States of America; University of California, San Diego, UNITED STATES

## Abstract

Invasion by infectious pathogens can elicit a range of cytokine responses from host cells. These cytokines provide the initial host defense mechanism. In this report, we demonstrate that TNF-α, a pro-inflammatory cytokine, can be induced by hepatitis C virus (HCV) in its host cells in a biphasic manner. The initial induction of TNF-α by HCV was prompt and could be blocked by the antibody directed against the HCV E2 envelope protein and by chemicals that inhibit endocytosis, indicating the specificity of endocytic uptake of HCV in this induction. Further studies indicated that the induction of TNF-α was dependent on toll-like receptors 7 and 8 (TLR7/8) but not on other intracellular pattern recognition receptors. Consistently, siRNA-mediated gene silencing of the downstream effectors in the TLR7/8 signaling pathway including MyD88, IRAK1, TRAF6, TAK1 and p65 NF-κB suppressed the expression of TNF-α. The role of p65 NF-κB in the induction of TNF-α via transcriptional up-regulation was further confirmed by the chromatin immunoprecipitation assay. TNF-α induced by HCV could activate its own receptor TNFR1 on hepatocytes to suppress HCV replication. This suppressive effect of TNF-α on HCV was due to its role in supporting interferon signaling, as the suppression of its expression led to the loss of IFNAR2 and impaired interferon signaling and the induction of interferon-stimulated genes. In conclusion, our results indicate that hepatocytes can sense HCV infection via TLR7/8 to induce the expression of TNF-α, which inhibits HCV replication via an autocrine mechanism to support interferon signaling.

## Author Summary

Hepatitis C virus (HCV) patients have increased levels of circulating tumor necrosis factor-α (TNF-α). In this report, we demonstrate that HCV can directly induce the expression of TNF-α in hepatocytes in a biphasic manner via NF-κB. The induction of TNF-α by HCV in the first phase is prompt, requires no HCV gene expression and is dependent on TLR7 and TLR8 and their downstream effectors. TNF-α induced by HCV supports interferon signaling via an autocrine mechanism and suppresses HCV replication, as abolishing the expression of TNF-α or its receptor TNFR1 results in the loss of IFNAR2, a subunit of the type I interferon receptor, and an increase of HCV replication. Our studies thus reveal an interesting interplay between HCV and hepatocytes, with the virus attempting to blunt the IFN response by depleting IFNAR2 and the host cell overcoming this blunting effect of HCV by using TNF-α to restore the expression of IFNAR2.

## Introduction

Hepatitis C virus (HCV) is an enveloped virus with a single-stranded RNA genome of 9.6-Kb [1]. After binding to its receptors on hepatocytes, HCV is internalized by receptor-mediated endocytosis, and its genomic RNA is subsequently released into the cytosol to direct the synthesis of viral proteins using the internal ribosome entry site (IRES) located near its 5’-end. This leads to the production of a polyprotein with a length of approximately 3000 amino acids. The HCV polyprotein is proteolytically cleaved by host and viral proteases to give rise to individual viral proteins including the core protein, E1 and E2 envelope proteins, the p7 viroporin, and nonstructural proteins NS2, NS3, NS4A, NS4B, NS5A, and NS5B [2].

Pattern recognition receptors (PRRs) including toll-like receptors (TLRs) and RIG-I-like receptors are important components of the innate immune response. Upon the activation by the pathogen-associated molecular patterns (PAMPs), these PRRs induce the expression of various cytokines via the downstream signaling pathways. Some TLRs are located on the cellular surface and sense extracellular PAMPs and some TLRs are located in the endosomes to detect internalized pathogens [3]. The TLR signaling is mediated by the TIR domain-containing cytosolic adaptors MyD88, TIRAP/Mal and TRIF. The initial association of MyD88 to the receptor leads to the sequential recruitment and activation of IRAK4 and IRAK1. The activated IRAK1 then binds to TRAF6, after which the complex dissociates from the receptor for further signaling events including the activation of TAK1. TAK1 can activate NF-κB and AP1 to stimulate the production of pro-inflammatory cytokines. IRAK1 and TRAF6 can also activate IRF7 to induce the expression of type I interferons (IFNs) [4, 5].

Tumor necrosis factor-α (TNF-α) is a pro-inflammatory cytokine produced in response to infectious pathogens. The soluble TNF-α is produced as a result of cleavage from its precursor transmembrane TNF-α by the TNF-α-converting enzyme (TACE). The secreted TNFα binds to its receptors, namely TNFR1 and TNFR2, to exert its biological effects [6]. Multiple studies indicate that the blood level of TNF-α is increased in HCV patients and its level is positively correlated with HCV pathogenesis and the severity of liver diseases [7–9]. The major source of TNF-α in response to HCV infection is unclear and thought to be immune cells such as T lymphocytes and macrophages [10, 11]. In this report, we provide evidence to demonstrate that hepatocytes can also produce TNF-α in response to HCV infection. This TNF-α induction is prompt and mediated by TLR7 and TLR8. Furthermore, we also demonstrate that TNF-α, through an autocrine mechanism, prevents the depletion of IFNAR2 by HCV and is required to support interferon signaling in HCV-infected cells.

## Results

### Induction of TNF-α by HCV in its host cells

**Fig 1:**
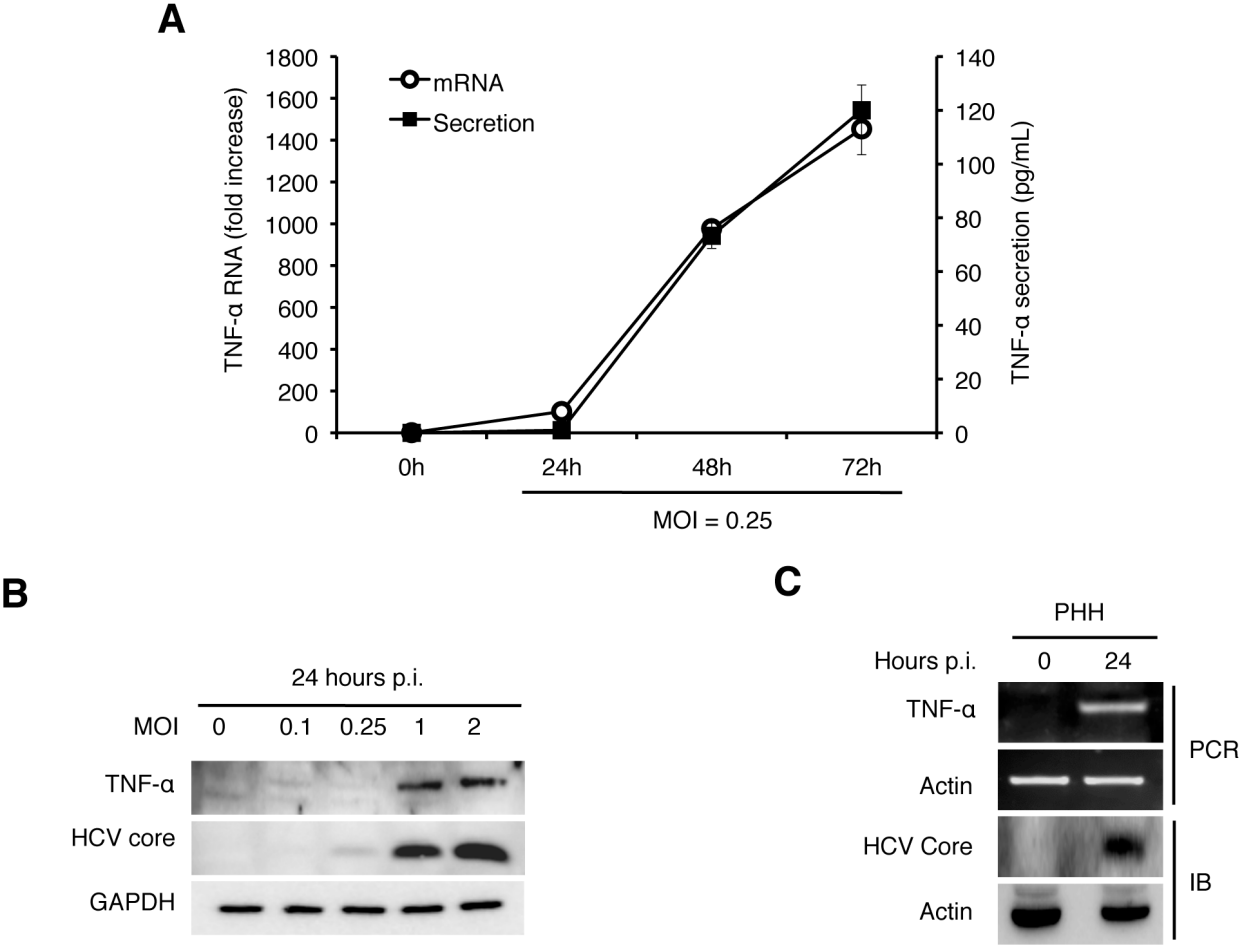
Induction of TNF-α by HCV. (A) Quantification of TNF-α in the culture media. Huh7 cells were infected with HCV (MOI = 0.25) and the culture media were collected at 0, 24, 48, and 72 hours post-infection. The level of TNF-α in the media was determined by ELISA and the TNF-α RNA in the cells were quantified by qRT-PCR. Bars indicate standard deviations calculated from three independent experiments. (B) Immunoblot analysis of TNF-α induction by HCV. Huh7 cells were infected with HCV using MOI of 0, 0.1, 0.25, 1 or 2 for 24 hours. Cells were then lysed for immunoblot analysis of TNF-α and the HCV core protein. GAPDH served as the loading control. (C) Induction of TNF-α in primary human hepatocytes (PHHs) by HCV. PHHs with or without HCV infection (MOI = 0.25) for 24 hours were lysed for analysis. Top two panels, semi-quantitative RT-PCR analysis of the TNF-α RNA and the actin RNA; bottom two panels, immunoblot analysis of the HCV core protein and α-actin.

To determine whether HCV infection can directly induce the expression of TNF-α in its host cells, we infected Huh7 hepatoma cells with a cell culture-adapted HCV JFH1 variant using a multiplicity of infection (MOI) of 0.25 and collected the incubation media at different time points after infection for quantification of TNF-α using ELISA. The soluble TNF-α was initially detectable at 48 hours post-infection and its level further increased at 72 hours (Fig 1A). When the quantitative RT-PCR (qRT-PCR) was used to analyze the expression of TNF-α RNA in cells, a similar induction profile was observed (Fig 1A), although a ~10-fold induction of TNF-α was also observed at 24 hours (see below). The induction of TNF-α in Huh7 cells could be detected by immunoblot at 24 hours post-infection when the MOI used was 1 or higher (Fig 1B). To ascertain that the TNF-α induction by HCV was not specific to Huh7 cells, we also infected primary human hepatocytes (PHH) with HCV. As shown in Fig 1C, HCV infection of PHH also induced the expression of TNF-α after 24 hours when a semi-quantitative RT-PCR was used for the analysis.

**Fig 2:**
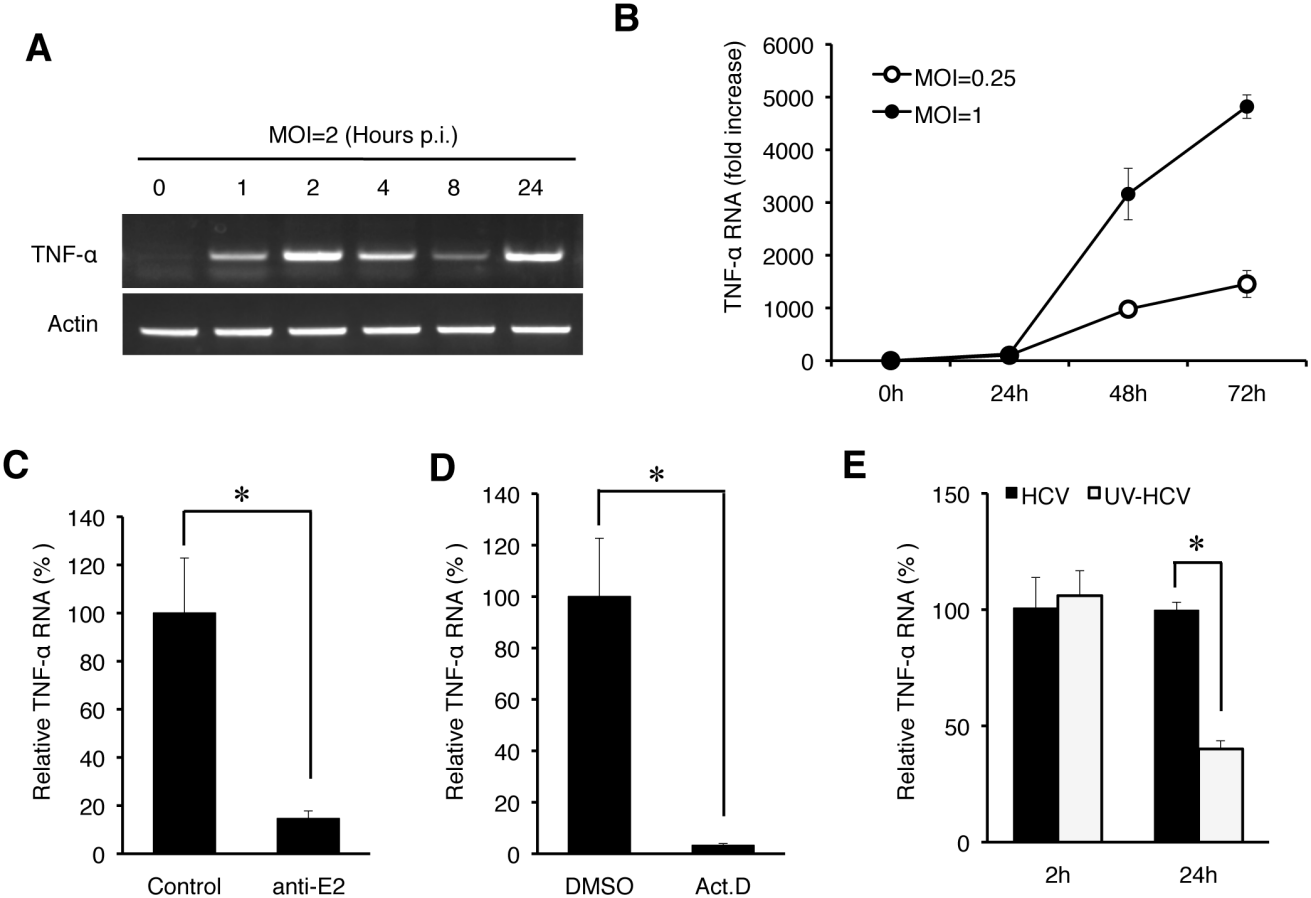
Induction analysis of TNF-α by HCV at early time points. (A) The time-course analysis of TNF-α induction by HCV. Huh7 cells were infected with HCV (MOI = 2) for 0, 1, 2, 4, 8, and 24 hours and then lysed for the isolation of total RNA, which was analyzed for TNF-α and actin RNAs by semi-quantitative RT-PCR. (B) The induction of TNF-α by HCV using the MOI of 0.25 and 1 was analyzed by qRT-PCR. (C) Huh7 cells were infected with HCV (MOI = 1) that had been pre-incubated with the isotype control antibody or the anti-E2 antibody at 4°C for 1 hour [13]. The total cellular RNA was isolated at 2 hours post-infection for qRT-PCR analysis of TNF-α RNA. TNF-α RNA induced by HCV that was treated with the isotype antibody control was arbitrarily defined as 100%. (D) Effect of actinomycin D on TNF-α induction by HCV. Huh7 cells were pretreated with DMSO or actinomycin D (5 μg/ ml) for 1 hour. Cells were then infected with HCV (MOI = 1) for 2 hours. Total cellular RNA was isolated for qRT-PCR analysis to assess TNF-α induction. TNF-α RNA induced by HCV in cells treated with DMSO was arbitrarily defined as 100%. (E) UV-inactivation of HCV. HCV (MOI = 1), with or without UV-irradiation for 5 minutes, was used to infect Huh7 cells. Cells were lysed at 2 hours and 24 hours post-infection for qRT-PCR analysis of TNF-α RNA. TNF-α RNA induced by HCV at 2 hours post-infection without UV treatment was arbitrarily defined as 100%. *, *p*<0.05.

To determine how early after infection HCV could induce the expression of TNF-α, we analyzed the induction of TNF-α by HCV within the first 24 hours of infection using an MOI of 2. As shown in Fig 2A, HCV could induce the expression of TNF-α as early as one hour post-infection, when the semi-quantitative RT-PCR was used for the analysis. This induction was increased at 2 hours, reduced at 4 and 8 hours and increased again at 24 hours (Fig 2A). We also used qRT-PCR to analyze the effect of MOI on the induction of TNF-α and found that the induction of TNF-α by HCV was slight at 2 hours but significant (~10-fold) at 24 hours post-infection when the MOI used was 0.25 (Fig 2B). However, when the MOI of 1 was used, the fold induction of TNF-α at 2 hours and 24 hours post-infection was similar at about 15, indicating a dose-effect of HCV on the induction of TNF-α at the early time points of infection. To rule out the possibility that the early induction of TNF-α was due to nonspecific factors in the HCV inoculum, we treated the HCV inoculum (MOI = 1) with an anti-E2 antibody, which neutralized the infectivity of HCV [12, 13]. As shown in Fig 2C, this neutralization antibody reduced the TNF-α RNA in Huh7 cells to almost the basal level, confirming the specificity of TNF-α induction by HCV. In addition, the early induction of TNF-α was inhibited, if cells were treated with actinomycin-D, an inhibitor of RNA synthesis, prior to HCV infection (Fig 2D), indicating a transcriptional up-regulation of TNF-α. The effect of actinomycin-D on TNF-α was unlikely due to its effect on HCV entry, as this treatment slightly increased the HCV RNA level in cells (S1 Fig). Considering that the induction of TNF-α occurred almost immediately after HCV infection, it did not appear likely that the translation or the replication of HCV genome RNA was involved. To test this possibility, we used UV-irradiation to inactivate HCV prior to infection. This UV inactivation did not inhibit the induction of TNF-α at two hours post infection (Fig 2E), indicating that the integrity of the HCV genome was not essential for the induction of TNF-α at this early time point. However, the UV-inactivation of HCV reduced the second-phase induction of TNF-α at 24 hours, indicating that the HCV gene expression and/or replication was required for the efficient induction of TNF-α at this later time point. Besides TNF-α, the induction of other cytokines including IL-6 and IL-1β was also observed in both Huh7 cells and PHH at 2 hours post-infection (S2 Fig).

### Endocytic uptake of HCV required for the induction of TNF-α

**Fig 3:**
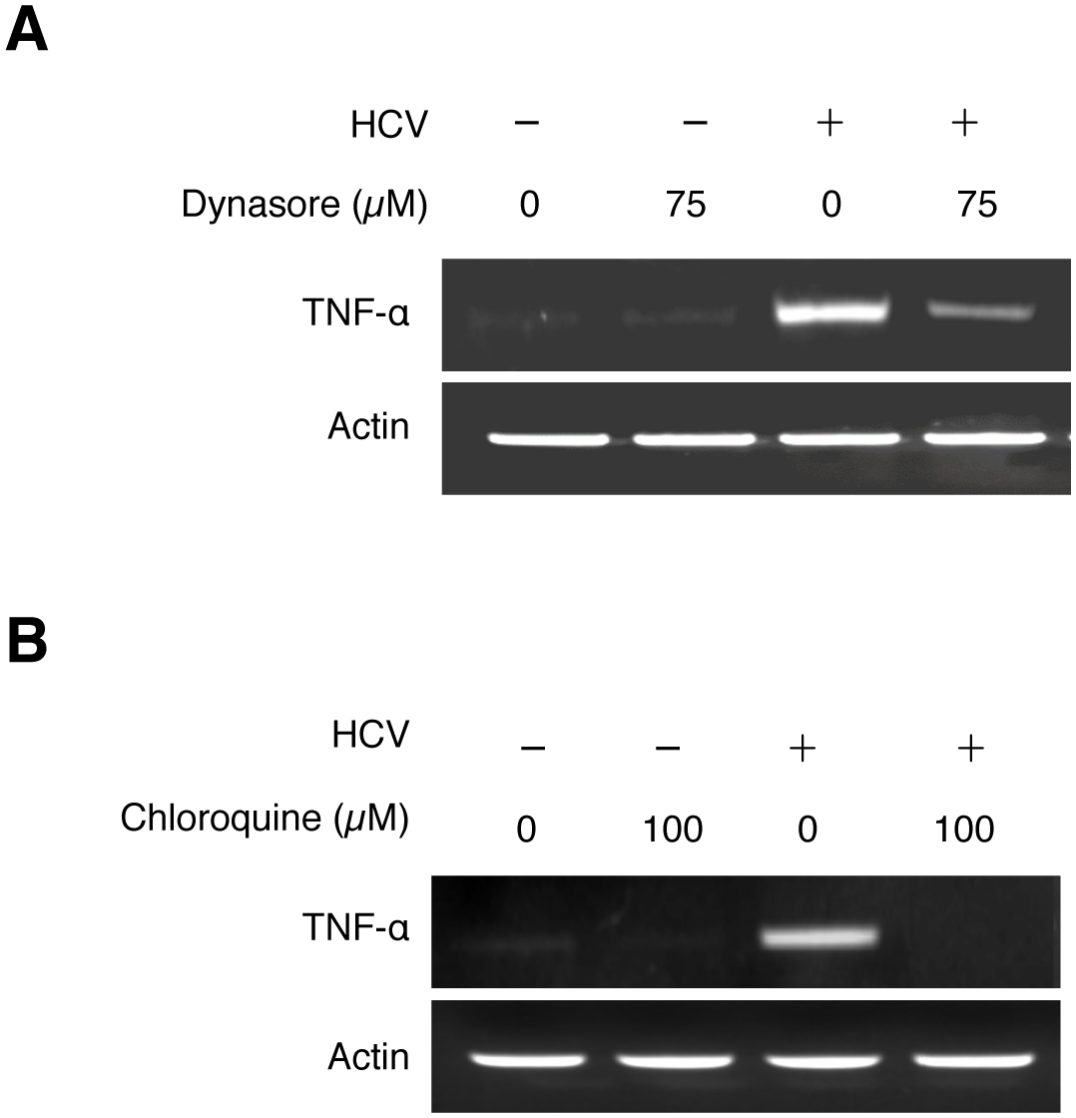
Induction of TNF-α by HCV is dependent on the endocytic pathway. Huh7 cells were pre-treated with 75 μM Dynasore (A), 100 μM Chloroquine (B), or the vehicle DMSO for 1 hour and during HCV infection (MOI = 0.25). Total cellular RNA was isolated at 2 hours post-infection for semi-quantitative RT-PCR analysis.

The finding that the induction of TNF-α was detected almost immediately after infection without the apparent need of an intact HCV genome suggested an early event of HCV infection in the induction of TNF-α, possibly during the viral entry. After binding to its co-receptors, HCV enters the cell via the clathrin-mediated endocytosis [1, 14]. To test whether this endocytic uptake is required for HCV to induce TNF-α, we treated Huh7 cells with Dynasore, a cell-permeable inhibitor of dynamin GTPase, which mediates the scission of clathrin-coated vesicles from plasma membranes. As shown in Fig 3A, the inhibition of dynamin with Dynasore significantly inhibited the induction of TNF-α by HCV. This result suggested an important role of endocytic uptake of HCV in the induction of TNF-α. After the dissociation of clathrin, endocytic vesicles fuse with endosomes. The acidic content of endosomes then triggers the fusion of HCV envelope with endosomal membranes for the release of the viral genome into the cytosol. To examine the possible importance of endosomal acidification in the induction of TNF-α, we treated cells with chloroquine, an inhibitor of endosomal acidification. As shown in Fig 3B, the treatment of Huh7 cells with chloroquine abolished the TNF-α induction by HCV. Altogether, these results suggested that both the scission of endocytic vesicles from plasma membranes and the endosomal acidification were important for the induction of TNF-α by HCV.

### Induction of TNF-α by HCV mediated by the TLR7/8 signaling pathway

RIG-I and MDA5 are two cytosolic PRRs that recognize double-stranded RNAs (dsRNAs), and the former has been shown to recognize the 3’-end UC-rich sequence of the HCV RNA [15]. To test the possible roles of these two PRRs in the induction of TNF-α by HCV, we performed the siRNA knockdown experiment to suppress the expression of these two proteins prior to HCV infection. As shown in S3A Fig, the suppression of RIG-I and MDA5 expression in Huh7 cells had only a marginal effect, if any, on the induction of TNF-α. The lack of effect of RIG-I on the induction of TNF-α by HCV was also confirmed by the infection of Huh7.5 cells with HCV. Huh7.5 cells were derived from Huh7 cells and expressed a defective RIG-I [16]. As shown in S3B Fig, HCV could induce TNF-α in Huh7.5 cells, confirming that RIG-I was not essential for the expression of TNF-α.

**Fig 4:**
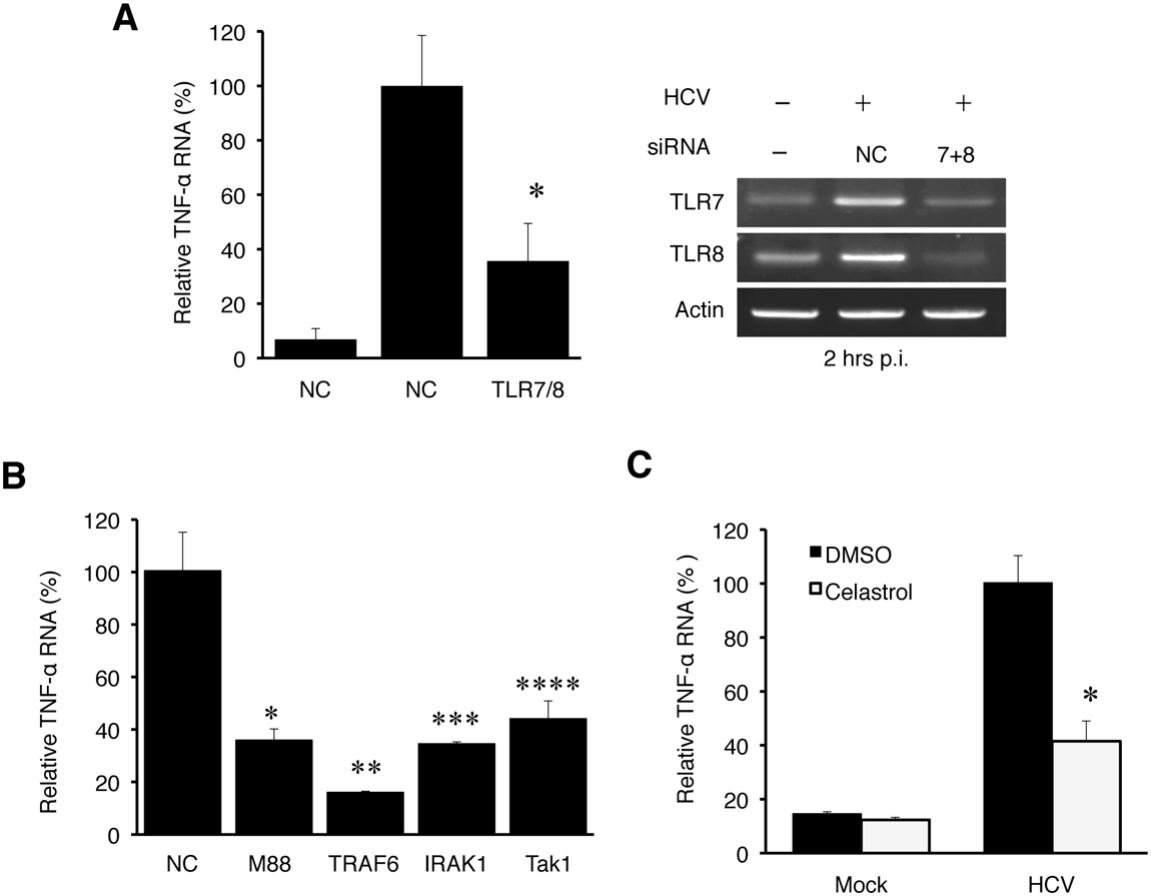
TNF-α induction by HCV is dependent on the TLR7 and TLR8 signaling pathway. (A) Huh7 cells were transfected with negative control (NC) siRNA or siRNAs targeting TLR7 and TLR8 for 6 hours, after which the siRNA complex was removed. At 48 hours post transfection, cells were mock-infected (-) or HCV-infected (+) (MOI = 1) for 2 hours. Total cellular RNA was then isolated and subjected to qRT-PCR for the analysis of TNF-α RNA (left panel). The knockdown efficiency of TLR7 and TLR8 was measured by semi-quantitative RT-PCR using actin RNA as a control (right panel). *, *p*<0.05. (B) Huh7 cells were transfected with negative control siRNA or siRNA targeting MyD88, IRAK1, TRAF6, or TAK1 for 6 hours. At 48 hours post transfection, cells were mock-infected or HCV-infected (MOI = 1) for 2 hours and then lysed for RT-PCR analysis. *, *p* = 0.0001; **, *p* = 0.014; ***, *p* = 0.004; ****, *p* = 0.013. The knockdown efficiency of these various factors is shown in S7 Fig. (C) Huh7 cells were pre-incubated with Celastrol with the concentrations indicated for 1 hour. Cells were then infected with HCV for 2 hours in the presence of the drug. Total cellular RNA was isolated for RT-PCR analysis of TNF-α RNA. *, *p*<0.05.

Due to the lack of significant effect of RIG-I and MDA5 on the induction of TNF-α by HCV, we turned our attention to TLR3, TLR7, TLR8 and TLR9, which are PRRs that reside in endosomes. Among them, TLR3 is activated by dsRNA; TLR7 and TLR8, which share a high degree of structural similarity and are functionally active in Huh7 cells (S4 Fig), are activated by single-stranded RNA (ssRNA); and TLR9 is activated by the unmethylated CpG motif of DNA [3]. To test the possible roles of TLR7 and TLR8 in the induction of TNF-α by HCV, we also performed the siRNA-knockdown experiments. HCV infection induced the expression of TNF-α at two hours post-infection and the simultaneous knockdown of both TLR7 and TLR8 (TLR7/8) impaired this induction (Fig 4A). It is not likely that the knockdown of TLR7/8 impaired HCV entry, as their simultaneous knockdown increased HCV RNA levels in cells at 24 hours post-infection (S5A Fig). The single knockdown of TLR7 or TLR8 had only a marginal effect on the induction of TNF-α (S5B Fig). This lack of significant effect of single knockdown of TLR7 or TLR8 on TNF-α was likely due to the compensatory increase of the expression of the other when the expression of either one of these two TLRs was suppressed (S5C Fig). In contrast to TLR7/8, the knockdown of TLR3 and TLR9 as well as TLR4, which recognizes lipopolysaccharides, had no apparent effect on the induction of TNF-α by HCV (S5D Fig). Note that HCV infection increased TLR7 and TLR8 RNA levels (Fig 4A and S5B Fig) and appeared to also slightly increase the TLR3 and TLR9 levels (S5D Fig). The reason for this is unclear, but the induction of TLR7 and TLR8 was apparently mediated by NF-κB, as the knockdown of p65, a subunit of NF-κB, largely abolished their induction by HCV (S6 Fig).

If HCV indeed induced the expression of TNF-α via TLR7 and TLR8, then the suppression of expression of their downstream adaptor molecules should also inhibit the induction of TNF-α by HCV. As shown in Fig 4B, the suppression of expression of MyD88, IRAK1, TRAF6 or TAK1, which mediates TLR7/8 signaling, all led to the reduction of TNF-α induction by HCV. The knockdown efficiency of these adaptor molecules was shown in S7 Fig. TAK1 activates the transcription factor NF-κB by phosphorylating IκB kinase β (IKKβ). Its role in the induction of TNF-α by HCV was further confirmed by the observation that Celastrol, an inhibitor of the TAK1 kinase, impaired the induction of TNF-α by HCV (Fig 4C). These adaptor molecules are not known to affect HCV entry and thus it is unlikely that their knockdown led to the reduction of TNF-α via the inhibition of HCV entry. Indeed, as shown in S8 Fig, the knockdown of TRAF6 did not decrease but rather increased the HCV RNA level. Taken together, our results demonstrated that HCV induced the expression of TNF-α via TLR7/8 and its downstream signaling molecules including MyD88, IRAK1, TRAF6, and TAK1.

### NF-κB-dependent induction of TNF-α by HCV

**Fig 5:**
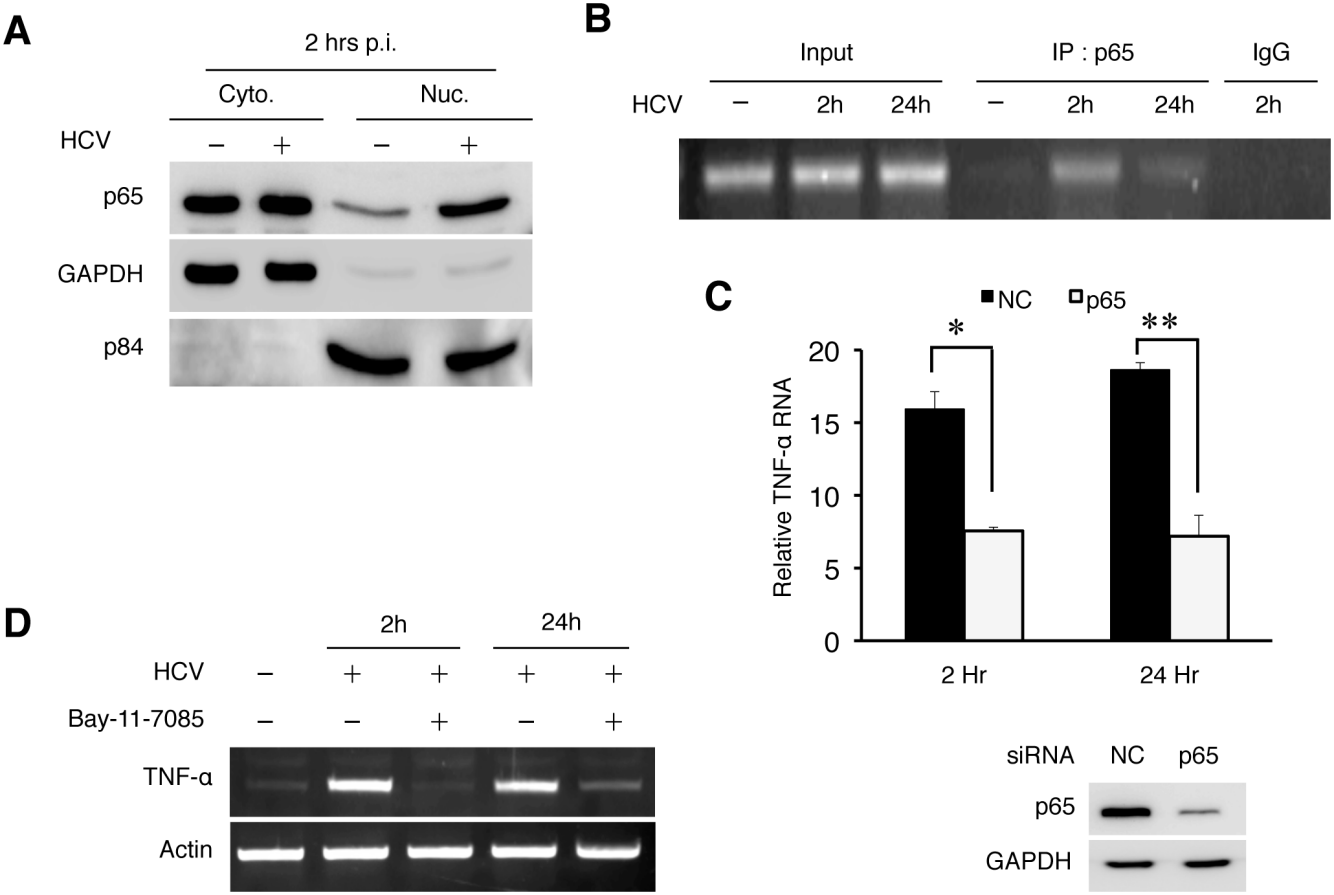
HCV-induced TNF-α by activating NF-κB. (A) Subcellular fractionation analysis of p65 NF-κB. Huh7 cells were mock-infected or HCV-infected (MOI = 1) for 2 hours and then subjected to subcellular fractionation as described in *Materials and Methods*. The subcellular localization of p65 NF-κB was then analyzed by immunoblot. GAPDH and the nuclear matrix protein p84 were used as markers to monitor the subcellular fractionation efficiency. (B) ChIP analysis for binding of NF-κB to the TNF-α promoter. Huh7 cells were infected with HCV (MOI = 1) for 2 or 24 hours followed by ChIP analysis using the anti-p65 antibody or a control IgG. Input, total TNF-α DNA without the immunoprecipitation. (C) p65 NF-κB knockdown experiment. Huh7 cells were transfected with the negative control (NC) siRNA or siRNA targeting p65 NF-κB followed by infection with HCV (MOI = 1) for 2 hours or 24 hours. Total cellular RNA was subjected to RT-PCR for analysis of TNF-α expression (left panel). The knockdown efficiency of p65 NF-κB was monitored by immunoblot, with GAPDH serving as the loading control (right panel). *, *p* = 0.0003, **, *p* = 0.002. (D) Effect of Bay-11-7085 on TNF-α expression. Huh7 cells were pre-treated with DMSO or 8μM Bay-11-7085 for 1 hour prior to infection with HCV (MOI = 1). Cells were then lysed at the time points indicated for semi-quantitative RT-PCR analysis of TNF-α RNA.

TAK1 activates IKKβ, which phosphorylates and destabilizes the NF-κB inhibitor IκB to result in the nuclear translocation of NF-κB. To determine whether NF-κB was indeed activated in the early time point of HCV infection, we performed the subcellular fractionation experiment. As shown in Fig 5A, HCV infection indeed induced the nuclear translocation of p65, a subunit of NF-κB, at 2 hours post-infection. To further test the role of NF-κB in the activation of the TNF-α gene, we performed the chromatin immunoprecipitation (ChIP) assay to determine the binding activity of p65 NF-κB to the TNF-α promoter, which contains the NF-κB binding site [17]. As shown in Fig 5B, an enhanced binding of p65 NF-κB to the TNF-α promoter was observed in HCV-infected cells at 2 hours post-infection. The binding of p65 NF-κB to the TNF-α promoter was also detected at 24 hours, albeit to a lesser degree. To further verify the role of p65 in TNF-α induction, we performed the p65 NF-κB knockdown experiment. As shown in Fig 5C, p65 NF-κB knockdown reduced the ability of HCV to induce the expression of TNF-α at both 2 hours and 24 hours post-infection. Consistently, Bay-11-7085, a chemical that inhibits the phosphorylation of IκBα and the activation of NF-κB, reduced the TNF-α expression (Fig 5D). Taken together, our results clearly demonstrated a role of NF-κB in the induction of TNF-α by HCV. Note that previous studies indicated that HCV could induce oxidative stress, which could activate NF-κB [18, 19]. However, it does not appear likely that oxidative stress was involved in the induction of TNF-α during the early time points of HCV infection, as the treatment of cells with the antioxidant N-acetylcysteine (NAC) had little effect on the induction of TNF-α by HCV at 2 hours and 48 hours post-infection (S9 Fig).

### Suppression of HCV replication by TNF-α via an autocrine mechanism

**Fig 6:**
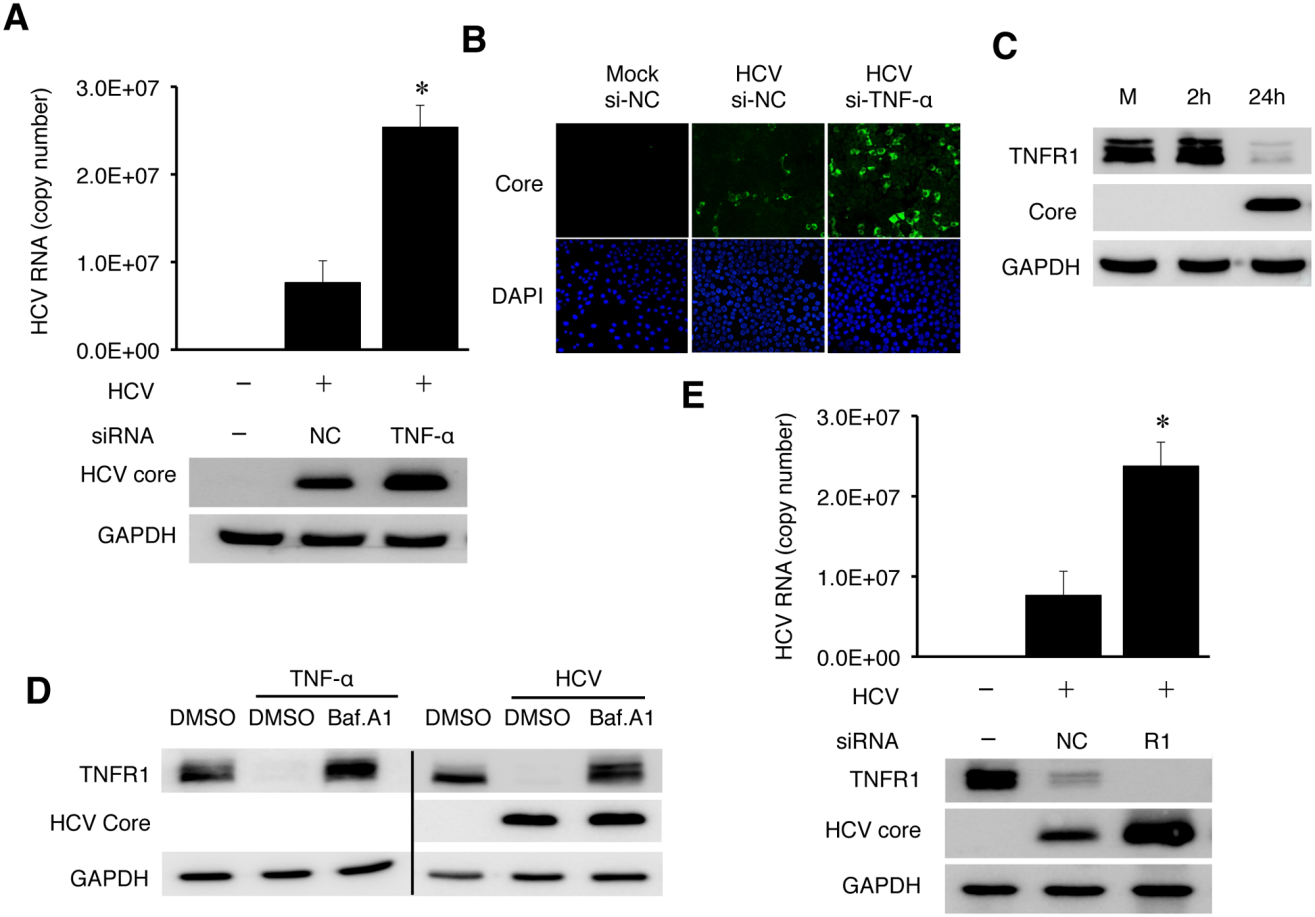
TNF-α knockdown enhanced HCV replication. (A-B) Huh7 cells were transfected with the negative control (NC) siRNA or TNF-α siRNA. After 48 hours, cells were transfected with the same set of siRNA for the second time for 6 hours before the HCV infection (MOI = 0.25) for 48 hours. The knockdown efficiency of TNF-α is shown in S12 Fig. (A) Real-time RT-PCR analysis of intracellular HCV RNA is shown in the histogram on the top. (Data are represented as mean ± SD). Immunoblot analysis of the HCV core protein and GAPDH are shown in the bottom two panels. (B) Immunofluorescence analysis of HCV titers. Naïve Huh7 cells were incubated for 20 hours with the conditioned media harvested from mock- or HCV-infected cells. Cells were then fixed and immunofluorescence stained for the HCV core protein. DAPI was used to stain nuclei. (C) Loss of TNFR1 in HCV-infected cells. Huh7 cells infected by HCV (MOI = 1) were lysed at the time points indicated and analyzed by immunoblot for TNFR1, HCV core protein and GAPDH. (D) Effect of Bafilomycin A1 (BafA1) on TNFR1. Huh7 cells without (left 3 lanes) or with HCV infection (MOI = 0.25) for 24 hours (right 3 lanes) were either not treated or treated with DMSO (D) or 200 nM Bafilomycin A1 (Baf) for additional 16 hours. Cells were then lysed for immunoblot analysis. GAPDH served as a loading control. (E) Effect of TNFR1 on HCV replication. Huh7 cells transfected with the control siRNA or the TNFR1 siRNA were infected with HCV (MOI = 0.25) and lysed at 48 hours post-infection for real-time RT-PCR analysis of HCV RNA (top panel, data represented as mean ± SD) and immunoblot analysis of TNFR1, the HCV core protein and GAPDH. *, *p*<0.05.

To determine whether TNF-α induced by HCV could directly affect HCV replication, we knocked down the expression of TNF-α using its specific siRNA prior to HCV infection. As shown in Fig 6A, the inhibition of TNF-α expression increased the levels of intracellular HCV RNA as well as the level of the HCV core protein, comparing with cells treated with the control siRNA. It also increased the HCV yield, as evidenced by the significant increase of HCV-positive cells when the progeny virus in the incubation media was harvested and used to infect naive cells (Fig 6B). To confirm that TNF-α could indeed suppress HCV replication, we also treated Huh7 cells with recombinant TNF-α, which also reduced the HCV RNA level (S10 Fig).

Soluble TNF-α exerts its effect through its receptors TNFR1 or TNFR2. TNFR1 is expressed in most cell types and believed to be responsible for most of the biological effects of TNF-α, while the expression of TNFR2 is primarily limited to endothelial cells and cells of hematopoietic lineages [20]. To understand how TNF-α exerted its inhibitory effect on HCV, we analyzed the expression of TNFR1 in HCV infected cells. As shown in Fig 6C, HCV caused the loss of TNFR1 at 24 hours post-infection. This loss of TNFR1 was a post-transcriptional event, as the TNFR1 RNA level was not affected by HCV (S11 Fig). As TNFR1, upon binding to TNF-α, is internalized by receptor-mediated endocytosis and degraded in lysosomes [21, 22], this result suggested an autocrine activation of TNFR1. To test this possibility, we first treated naive Huh7 cells with TNF-α. This treatment indeed caused the loss of TNFR1, which could be restored if cells were treated with Bafilomycin-A1 (BafA1) (Fig 6D), which inhibits the vacuolar ATPase activity and endocytic protein degradation in lysosomes [23]. We then treated HCV-infected cells at 24 hours post-infection with BafA1. As shown in the same figure, BafA1 also restored the TNFR1 protein level in HCV-infected cells. These results were consistent with the degradation of TNFR1 in lysosomes. Similar to the TNF-α depletion, silencing TNFR1 with its siRNA also increased HCV RNA and core protein levels (Fig 6E). These results strongly supported the argument that TNF-α suppressed HCV replication via an autocrine mechanism.

### Depletion of IFNAR2 by HCV when the expression of TNF-α or TNFR1 was inhibited

**Fig 7:**
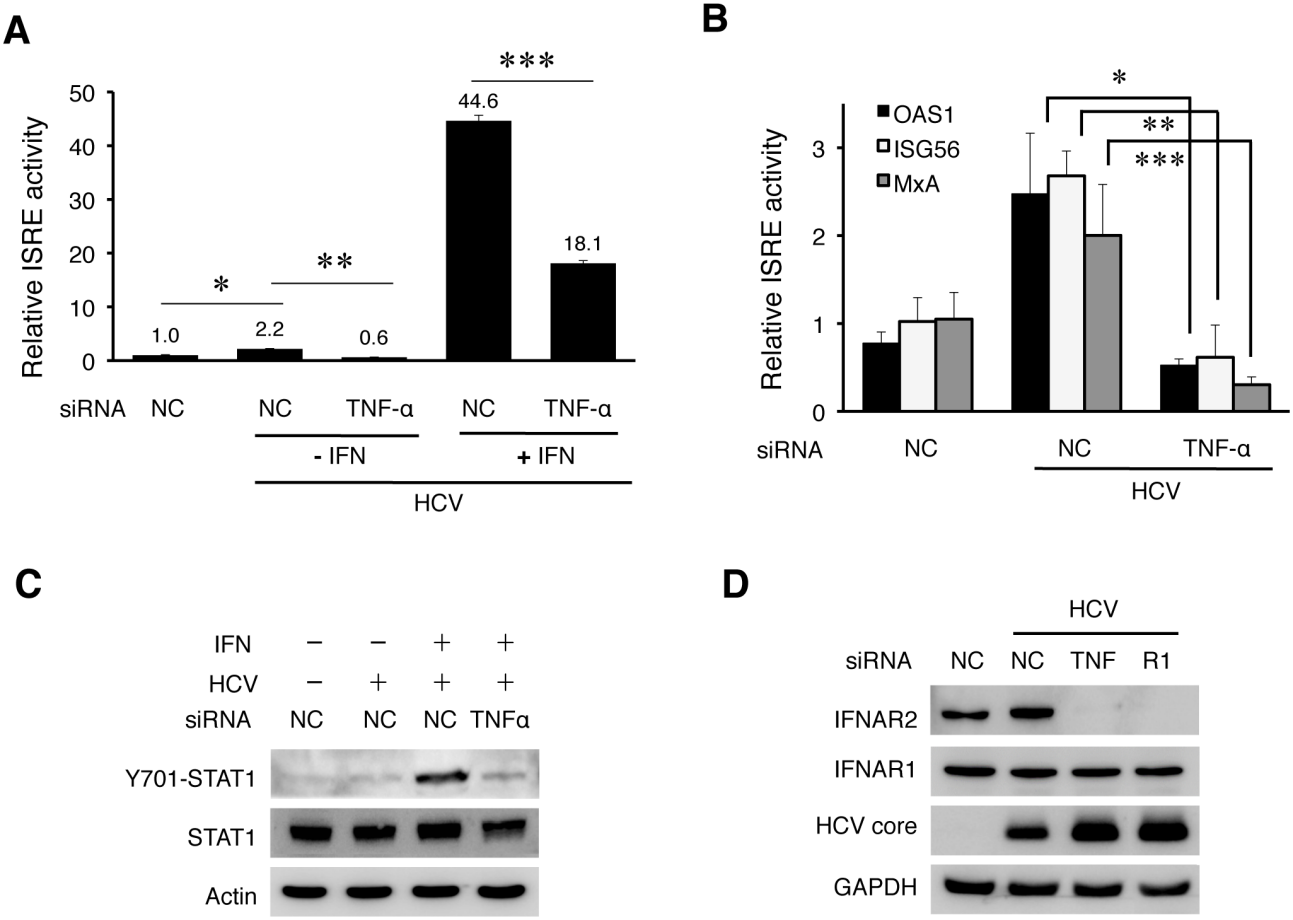
TNF-α induced by HCV is required to support IFN signaling. (A) Analysis of the ISRE activity. Huh7 cells were transfected with the control siRNA (NC) or the TNF-α siRNA. After 48 hours, cells were transfected with the NC or TNF-α siRNA again with the ISRE-firefly luciferase reporter plasmid and the pRL-SV40 plasmid. The pRL-SV40 plasmid (Promega) expressed the renilla luciferase reporter and was used as the internal control to monitor the transfection efficiency. Cells were infected with HCV (MOI = 0.25) for 6 hours the next day after transfection and then further incubated in fresh media with or without IFN-α (1000 units) for additional 18 hours. The activity of ISRE was measured by the dual luciferase assay. The Y-axis indicates the relative ISRE activity with the activity of mock-infected cells arbitrarily defined as 1. The results represented the average of three independent experiments. The knockdown efficiency of TNF-α is shown in S14A Fig. Bars indicate standard deviations calculated from three independent experiments, and asterisks indicate statistical significance with the p-values of 8.16E-05 (*), 5.37E-06 (**), and 2.37E-06 (***). (B) Inhibition of the induction of ISGs by depletion of TNF-α. Huh7 cells transfected with the negative control (NC) or the TNF-α siRNA were infected with HCV (MOI = 0.25) for 48 hours. Cells were then lysed for the analysis of various ISG RNAs by qRT-PCR. *, *p* = 0.0006, **, *p* = 0.007, ***, *p* = 0.04. (C) Immunoblot analysis of STAT1. Huh7 cells were transfected with the control or the TNF-α siRNA and infected with HCV (MOI = 0.25) followed by IFN-α treatment as in (A). Cells were then lysed for immunoblot analysis for phosphorylated STAT1, total STAT1, HCV core protein and actin. The knockdown efficiency of TNF-α was monitored by RT-PCR. (D) Effect of TNF-α and TNFR1 on the expression of IFNARs. Huh7 cells were transfected with the control siRNA, TNF-α siRNA or the TNFR1 siRNA and 48 hours later infected with HCV (MOI = 0.25). Mock-infected cells that were transfected with the control siRNA were used as the control. Cells were lysed at 48 hours post-infection for immunoblot analysis.

TNF-α unlikely suppressed HCV replication via the induction of apoptosis, as the knockdown of its expression using the siRNA enhanced, rather than suppressed, apoptosis of HCV-infected cells (S13 Fig). HCV infection can induce a modest level of type I interferons, which stimulate the expression of interferon stimulated genes (ISGs) [24–26]. To understand how TNF-α suppressed HCV replication, we analyzed the possible effect of TNF-α on IFN signaling in HCV-infected cells using a firefly luciferase reporter linked to the interferon-stimulated response element (ISRE). As shown in Fig 7A, in agreement with the previous reports [24–26], HCV infection slightly increased the ISRE activity and this increase was reduced to below the background level when TNF-α was depleted with the siRNA. The treatment of HCV-infected cells with IFN-α significantly increased the ISRE activity. Similarly, this increase was impaired, if TNF-α was depleted. The effect of TNF-α on ISRE was confirmed by analyzing the expression of OAS1, ISG56 and MxA, three IFN-stimulated genes (ISGs). As shown in Fig 7B, HCV could also increase the expression of these ISGs in Huh7 cells. This result was consistent with the previous reports that HCV could induce a low level of interferon response [24–26]. However, this induction was largely abolished, if the expression of TNF-α was inhibited with the siRNA. These results indicated that TNF-α, produced by the cells in response to HCV infection, was required to support interferon signaling and the expression of ISGs in HCV-infected cells. This is likely how TNF-α suppressed HCV replication.

Type I IFNs bind to the IFN-α receptor (IFNAR), which is composed of two subunits IFNAR1 and IFNAR2. This binding activates Janus kinase 1 (JAK1) and tyrosine kinase 2 (TYK2), which then phosphorylate and activate STAT1 and STAT2 to result in the activation of ISRE in the promoters of ISGs. To understand why TNF-α was required to support the expression of IFNs and ISGs, we analyzed the effect of TNF-α on the activation of STAT1 and STAT2. As shown in S14B Fig, HCV did not apparently affect the phosphorylation of STAT1 and STAT2 at 24 hours post-infection, but it slightly increased the STAT1 phosphorylation at 48 hours post-infection, in agreement with its modest effect on ISRE. The phosphorylation of both STAT1 and STAT2 was clearly visible after the treatment with IFN-α. Interestingly, when TNF-α was depleted with its siRNA, the phosphorylation of STAT1 induced by IFN-α was significantly inhibited in HCV-infected cells (Fig 7C). TNF-α could also enhance the phosphorylation of STAT1 and the expression of ISGs induced by IFN-α in naive Huh7 cells (S14C Fig). To further investigate why the activation of STAT1 by IFN-α was inhibited when TNF-α was depleted, we analyzed the expression levels of IFNAR1 and IFNAR2 in HCV-infected cells. As shown in Fig 7D, the depletion of either TNF-α or TNFR1 led to the loss of IFNAR2, but not IFNAR1, in HCV-infected cells. This loss of IFNAR2 was a post-transcriptional event, as the level of IFNAR2 mRNA was not apparently affected by the depletion of TNF-α or TNFR1 (S14D Fig), and most likely mediated by proteasomes, as its loss could be inhibited by the proteasome inhibitor MG132 but not by Bafilomycin A1 (S15 Fig).

Taken together, the results shown in Fig 7 indicated that TNF-α induced by HCV was required to maintain the IFNAR2 expression level to support IFN signaling. In the absence of TNF-α, IFNAR2 was depleted by HCV and IFN signaling was impaired.

## Discussion

HCV patients have an elevated serum level of TNF-α, and this level is positively correlated with the severity of liver diseases [7–9]. The source of TNF-α is unclear, but it is generally assumed that it is produced by immune cells such as macrophages [27]. In this report, we demonstrated that TNF-α could also be induced in HCV-infected cells. Although the amount of TNF-α produced by HCV-infected hepatocytes might be lower than that produced by professional immune cells such as macrophages [28], it was sufficient to trigger an inhibitory response on HCV replication. Our finding is consistent with a previous report, which described an increased level of TNF-α in the hepatocytes of HCV patients [9]. The induction of TNF-α by HCV was specific, as it could be blocked by the antibody that neutralized the infectivity of HCV (Fig 2C). This induction was biphasic, with the first phase of induction peaked at 2 hours post-infection (Fig 2A). The induction of TNF-α in the first phase was dependent on TLR7/8 (Fig 4A) and required no HCV gene expression or replication (Fig 2E). As TLR7 and TLR8 are activated by ssRNA, HCV either has to release the viral genomic RNA into endosomes during endocytosis to activate TLR7/8 or the HCV genomic RNA released into the cytosol after uncoating must be delivered immediately back into the endosomes. We favor the first scenario, as, if HCV RNA is released first into the cytosol, then it will likely also activate RIG-I and/or MDA5, which are cytosolic PRRs. However, we found that these two PRRs did not play a significant role in the induction of TNF-α (S3A Fig). If HCV indeed activates TLR7/8 during endocytosis, then the HCV virion must be disintegrated during this process for the genomic RNA to be released. This may be triggered by the acidic content of endosomes/lysosomes, which may destabilize HCV virion to release the viral RNA.

The activation of the TLR7/8 signaling pathway by HCV led to the activation of NF-κB (Fig 5A). This pathway was required for the induction of TNF-α by HCV in the first phase. The induction of TNF-α in the second phase also required NF-κB, as the depletion of p65 NF-κB also suppressed the second-phase induction of TNF-α by HCV (Fig 5C). It does not appear likely that this second-phase induction of TNF-α was due to the second-round of infection by progeny virus particles, as this second-phase induction of TNF-α was long-lasting (Fig 1A). A number of factors had been shown in the past to activate NF-κB in HCV-infected cells. These factors include TLR3 and protein kinase R (PKR), which could both be activated by the double-stranded HCV RNA replicative intermediates [29, 30]. These factors are likely the reasons why HCV was also able to induce TNF-α in the later phase of infection.

TNF-α induced by HCV suppressed HCV replication (Fig 6A). Our results indicated that this was likely due to its role in interferon signaling and the induction of ISGs (Fig 7A and 7B). We found that both TNF-α and TNFR1 participated in IFN signaling by maintaining the stability of IFNAR2, as in the absence of either one of them, IFNAR2 was lost in HCV-infected cells (Fig 7D), apparently due to degradation by proteasomes (S15 Fig). How HCV induced the degradation of IFNAR2 and how TNF-α antagonized this effect of HCV are interesting questions that remain to be determined. It is noteworthy that the degradation of IFNAR1 and IFNAR2 had previously been shown to be regulated by different mechanisms [31], and thus the selective degradation of IFNAR2 by HCV without affecting IFNAR1 was not unexpected. Nevertheless, our results reveal an interesting interplay between the virus and the host cell, with the virus attempting to blunt the IFN response by depleting IFNAR2 and the host cell overcoming this blunting effect of HCV by using TNF-α to restore the expression of IFNAR2.

Although our results indicated that TNF-α could support IFN signaling to suppress HCV replication in cell cultures, the role of TNF-α in HCV replication and pathogenesis *in vivo* may be more complicated. This is due in part to our observation that TNF-α suppressed apoptosis of HCV-infected cells (S13 Fig), which would favor HCV persistence, in part to the pro-inflammatory activities of this cytokine, and in part to a recent report that TNF-α could depolarize liver cells to enhance HCV entry [28]. Thus, it is tempting to speculate that TNF-α induced in the first phase may enhance HCV entry whereas it induced in the second phase may suppress HCV replication. This may explain why in the clinical trial of a limited number of HCV patients, the TNF-α inhibitor Etanercept was found to improve the therapeutic effect of IFN-α and ribavirin on HCV rather than to suppress it [32].

## Materials and Methods

### Cell cultures and HCV preparation

Huh7 cells were maintained in Dulbecco’s modified eagle medium (DMEM) supplemented with 10% fetal bovine serum (FBS). Huh7.5 cells were maintained in DMEM supplemented with 10% FBS and 1% nonessential amino acids. Primary human hepatocytes (PHHs) were obtained from the Cell Culture Core Facility of the USC Research Center for Liver Diseases. They were maintained in DMEM medium supplemented with what10% FBS. The JFH1 (HCV genotype 2a) variant, which produced a high level of infectious virus particles [33], was propagated in Huh7.5 and used in our infection studies.

### Measurement of TNF-α secretion

Huh7 cells were infected with HCV with an MOI of 0.25, and the incubation medium was replaced with the fresh medium after 3 hours of infection. The incubation medium was collected at the time points indicated and TNF-α was assayed using the human TNF-α Instant ELISA kit (eBioscience) following the instructions of the manufacturer.

### Immunoblot analysis and antibodies

Cells were lysed in M-PER Mammalian Protein Extraction Reagent (Thermo scientific) containing the protease inhibitor cocktail, 1mM PMSF, 1mM sodium orthovanadate, and 1mM sodium fluoride for 10 minutes on ice followed by a brief sonication. Cell lysates were cleared by centrifugation at 14,000 x g for 2 minutes. The supernatant was collected, boiled in Laemmli buffer for 5 minutes, and used for immunoblot analysis or stored at −80°C for future use. The rabbit anti-HCV core antibody was prepared in our laboratory [34]. TNFR1, p65 NF-κB, PARP, Caspase-8, and IRAK1 antibodies were from Cell signaling, and actin and IFNAR2 antibodies were from Sigma. IFNAR1 antibody was from Abcam, and GAPDH, TLR7, TLR8, and TRAF6 antibodies were from Santa Cruz. Horseradish peroxidase (HRP)-conjugated goat anti-rabbit and rabbit anti-mouse secondary antibodies were also purchased from Abcam.

### Gene silencing and generation of stable cell line

siRNAs targeting TNF-α, TNFR1, TLR3, TLR4, TLR7, p65 NF-κB, MyD88, IRAK1 and TAK1 were from Sigma. siRNAs targeting TLR7, TLR8, MDA5 and RIG-I were from Qiagen, and the siRNA targeting TRAF6 was from Santa Cruz. The transfection of siRNA into Huh7 cells was performed using Lipofectamine RNAiMax (Invitrogen). Briefly, Huh7 cells seeded in 100 mm dishes were transfected with 600 pmole siRNA for 6 hours. The transfected cells were further incubated in fresh media for 48 hours prior to infection with HCV. For generation of stable cell line, lentiviral particles were first produced in 293T cells through the coexpression of pLKO.TRC plasmid with shRNA insertion that targets TLR7 or TLR8 and packaging vectors. Lentiviral particles were harvested 60 hours post transfection and filtered. Huh7 cells were then infected with these lentiviral particles and selected with puromycin (2 μg/mL). Protein lysates were extracted 10 days post selection and immunoblotted with antibodies against TLR7 and TLR8.

### Semi-quantitative RT-PCR

Total cellular RNA was isolated using TRIzol (Invitrogen) and reverse-transcribed to cDNA using SuperScript II First-Strand Synthesis System for RT-PCR (Invitrogen). The cDNA was then subjected to PCR amplification with the primer sets listed in the table in the supplemental information (S1 Table).

### Quantitative reverse transcription polymerase chain reaction (qRT-PCR)

For the quantification of HCV RNA, total cellular RNA was subjected to qRT-PCR using the TaqMan EZ RT-PCR Kit (Applied Biosystems, Foster City, CA) following the manufacturer’s instructions. HCV JFH1 primers 5′-TCTGCGGAACCGGTGAGTA-3′ (forward) and 5′-TCAGGCAGTACCACAAGGC-3′ (reverse) and the probe 5′-CACTCTATGCCCGGC

CATTTGG-3′ were used for the qRT-PCR. The control GAPDH primer set with the probe was purchased from Applied Biosystems. For detection of other gene expressions, 100 ng total RNA was analyzed using the *Power* SYBR Green RNA-to-CT 1-Step Kit (Applied Biosystems, Foster City, CA). The primers used are shown in S1 Table.

### Subcellular fractionation

Huh7 cells with or without HCV infection were rinsed once with phosphate-buffered saline (PBS) and then trypsinized. Cells were washed once more with PBS before the subcellular fractionation using NE-PER Nuclear and Cytoplasmic Extraction Reagents (Thermo Scientific) following the manufacturer’s instructions.

### Chromatin immunoprecipitation (ChIP)

Binding of p65 NF-κB to the TNF-α promoter was analyzed using the Abcam ChIP kit following the manufacturer’s instructions. Briefly, Huh7 cells were infected with HCV for the indicated time period. After which, 1 x 10^6^ cells were fixed with 4% formaldehyde for 10 minutes at the room temperature. Cells were then lysed and the chromatin was sheared by sonication for 10 minutes. The chromatin was then immunoprecipitated using the anti-p65 antibody or a control IgG. The immunoprecipitate DNA samples were analyzed for the TNF-α promoter by PCR.

## Supporting Information

S1 Fig The effect of actinomycin-D on HCV replication. Huh7 cells that had been pretreated with DMSO or actinomycin D for 1 hour were infected with HCV (MOI = 1) in the presence of DMSO or actinomycin D. After 2 hours of infection, the HCV inoculum was removed and cells were further incubated in fresh media for 8 hours. For the qRT-PCR analysis of HCV RNA, 100 ng total RNA was used. *, *p*<0.05.
(TIFF)

S2 Fig Induction of inflammatory cytokines by HCV in Huh7 cells and PHHs. Huh7 cells or PHH were infected with HCV (MOI = 1) for 2 hours. Total cellular RNA was then isolated and analyzed by semi-quantitative RT-PCR for TNF-α, IL-6 and IL-1β RNAs.
(TIFF)

S3 Fig Analysis of various PRRs on the induction of TNF-α by HCV. (A) Huh7 cells were transfected with the negative control (NC) siRNA or the siRNA directed against RIG-I or MDA5. At 48 hours post transfection, cells were infected with HCV (MOI = 1) for 2 hours. An equal amount of cellular RNA was subjected to RT-PCR for analysis of TNF-α induction and the knockdown efficiency of RIG-I and MDA5. (B) Huh7.5 cells were infected with HCV (MOI = 1) for 2 hours. Cells were then lysed for the RT-PCR analysis of TNF-α induction by HCV.
(TIFF)

S4 Fig Induction of TNF-α by TLR7/8 agonist R848 in naïve Huh7 cells. Huh7 cells were treated with 0, 25 or 50 μg/ml imidazoquinoline resiquimod (i.e., R848, the TLR7/8 agonist) for 24 hours. Total cellular RNA was analyzed for TNF-α RNA by qRT-PCR using GAPDH RNA as the control. * and **, *p*<0.05.
(TIFF)

S5 Fig Relationship between TLRs and HCV infection. (A) Huh7 cells were transfected with NC siRNA or siRNAs targeting TLR7 and TLR8 for 6 hours, after which the siRNA complex was removed. At 48 hours post transfection, cells were mock-infected (-) or HCV-infected (+) (MOI = 1) for 24 hours. Total cellular RNA was then isolated for qRT-PCR analysis of HCV RNA. *, *p*<0.05. (B) Huh 7 cells were transfected with the control siRNA or siRNA targeting TLR7 or TLR8. After 48 hours, cells were infected with HCV (MOI = 1). Cells were lysed at 2 hours post-infection for RT-PCR analysis of various RNAs. (C) 293T cells were co-transfected with the pLKO.TRC plasmid (Addgene) that expressed TLR7 or TLR8 shRNA and the packaging vector (Addgene) for the production of recombinant lentiviral particles, which were harvested 60 hours post-transfection. Huh7 cells were then infected with these lentiviral particles and selected with puromycin (2μg/mL). Protein lysates were extracted from cell colonies 10 days post-selection and immunoblotted with antibodies against TLR7 and TLR8. shTLR7-1 and shTLR7-3 were two different shRNAs that targeted TLR7. Similarly, shTLR8-1 and shTLR8-3 were two different shRNAs that targeted TLR8. (D) The experiments were conducted using the same procedures mentioned above in (B), with the exception that TLR3, TLR4 and TLR9 siRNAs were used.
(TIFF)

S6 Fig p65 NFκB-mediated up-regulation of TLR7 and TLR8 expression. Huh 7 cells were transfected with the control siRNA or siRNA targeting p65 NFκB. At 48 hours after transfection, cells were infected with HCV (MOI = 1) for 2 hours. Total RNA was analyzed for TLR7 and TLR8 RNA expression using qRT-PCR. RNAs. The immunoblot of p65 was shown to the right for monitoring its knockdown efficiency. * and **, *p*<0.05.
(TIFF)

S7 Fig Analysis of the knockdown efficiency of various TLR signaling factors. Huh7 cells were transfected with the negative control (NC) siRNA or siRNA targeting (A) MyD88, (B) IRAK1, (C) TRAF6, or (D) TAK1 two days prior to HCV infection. The transfected cells were then mock-infected or infected with HCV for 2 hours before being lysed for analysis. The knockdown efficiency of MyD88, IRAK1, and TAK1 was analyzed by immunoblotting (A, B, and D) and that of TRAF6 was analyzed by RT-PCR (C).
(TIFF)

S8 Fig The effect of TRAF6 knockdown on HCV replication. Huh7 cells were transfected with NC siRNA or siRNAs targeting TRAF6. Forty-eight hours post-transfection, cells were mock-infected (-) or HCV-infected (+) (MOI = 1) for 24 hours. Total cellular RNA was then isolated for qRT-PCR analysis of TNF-α RNA. *, *p*<0.05.
(TIFF)

S9 Fig The effect of NAC on TNF-α induction by HCV. Huh7 cells were pretreated with NAC for one hour prior to infection with HCV (MOI = 1) and for another two hours after HCV infection, or with NAC during the final six hours of the 48-hour infection. Total RNA was isolated for RT-PCR analysis of TNF-α RNA.
(TIFF)

S10 Fig The effect of TNF-α on HCV replication. Huh7 cells were pretreated with recombinant human TNF-α (R&D systems) for 4 hours prior to and during infection with HCV (MOI = 1). Total cellular RNA was isolated at 20 hours post-infection for qRT-PCR analysis of HCV RNA. * and **, *p*<0.05.
(TIFF)

S11 Fig Analysis of TNFR1 RNA in Huh7 cells with or without HCV infection. Huh7 cells with or without HCV infection for 48 hours were lysed for the isolation of total RNA, which was then analyzed for TNFR1 RNA by semi-quantitative RT-PCR.
(TIFF)

S12 Fig Analysis of the knockdown efficiency of TNF-α. The experiments were conducted as described in the legend of Fig 6A and 6B. The knockdown efficiency of TNF-α was analyzed by semi-quantitative RT-PCR.
(TIFF)

S13 Fig The effect of TNF-α depletion on apoptosis. Huh7 cells were transfected with negative control (NC) siRNA or TNF-α siRNA. At 48 hours post transfection, cells were mock-infected or HCV-infected (MOI = 0.25) for 48 hours. Cells were then lysed for immunoblot analysis of poly (ADP-ribose) polymerase (PARP) and caspase 8. GAPDH was used as an internal loading control. The locations of cleaved PARP, procaspase 8 (Pro) and cleaved fragments (CF) of caspase 8 are indicated. Note that the knockdown of TNF-α enhanced apoptosis, as evidenced by the enhanced cleavage of PARP and procaspase 8, which are markers of apoptosis.
(TIFF)

S14 Fig Effects of TNF-α on interferon signaling. (A) Analysis of the knockdown efficiency of TNF-α, which was analyzed by semi-quantitative RT-PCR. The actin RNA was also analyzed to serve as the internal control. This study is to support Fig 7A. (B) Huh7 cells were infected with HCV for either 4 hours or 30 hours and then further incubated with or without IFN-α (1000 units) for 18 hours. Cells were then lysed at either 24 hours or 48 hours post-infection for immunoblot analysis of the phosphorylation status of STAT1 and STAT2. Total STAT1 and STAT2 and the HCV core expression were also analyzed. Actin served as the loading control. (C) Huh7 cells were incubated with IFN-α in absence or presence of TNF-α for 24 hours. STAT1 phosphorylation was analyzed by western blot, and relative ISG expression levels were analyzed by qRT-PCR. *, *p* = 0.01, **, *p* = 8.5E-05, ***, *p* = 0.005. (D) Huh7 cells were transfected with NC-, TNF-α-, or TNFR1-siRNA. After 48 hours, the siRNA transfection was repeated. Cells were infected with HCV at 6 hours after the second siRNA transfection. Total cellular RNA was isolated at 48 hours post-infection and analyzed by semi-quantitative RT-PCR for IFNAR1 and IFNAR2 RNAs. The actin RNA was also analyzed to serve as the control.
(TIFF)

S15 Fig Proteasome-mediated degradation of IFNAR2 in the TNF-α depleted cells. Huh7 cells were transfected with the negative control siRNA or TNF-α siRNA. After 48 hours, cells were infected with HCV (MOI = 0.25) for 24 hours and then treated with DMSO, 200 nM Bafilomycin A1, or 10 μM MG132 for additional 16 hours. Cells were then lysed for immunoblot analysis of IFNAR2 and the HCV core protein. GAPDH served as the loading control.
(TIFF)

S1 Table List of PCR primers. (TIFF)
