# Supplementary figures and images for: TNF-α Induced by Hepatitis C Virus via TLR7 and TLR8 in Hepatocytes Supports Interferon Signaling via an Autocrine Mechanism
Source: PLoS Pathog. 2015 May 29;11(5):e1004937. doi: 10.1371/journal.ppat.1004937 (PMC4449221; doi:10.1371/journal.ppat.1004937)

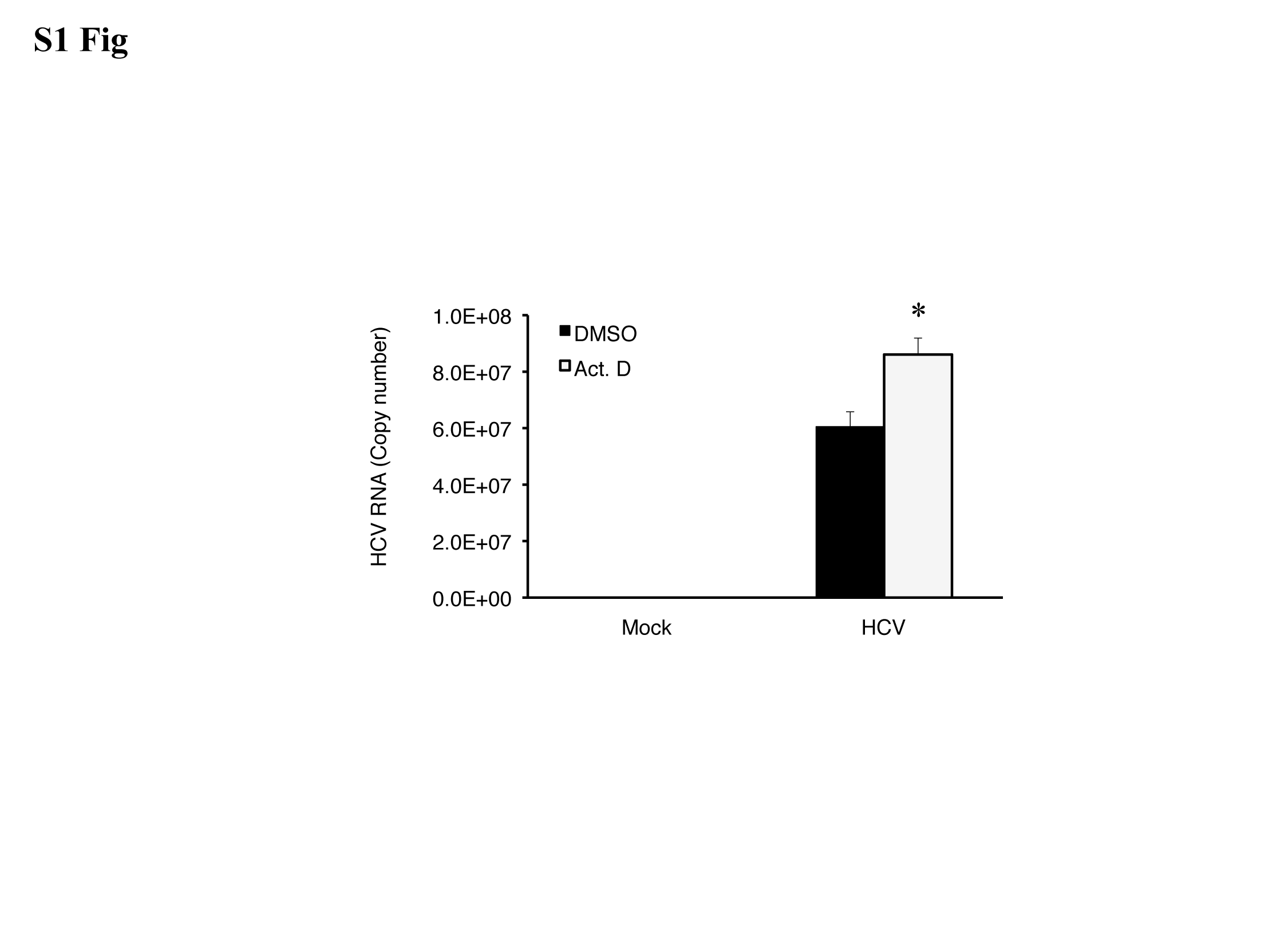

Supplement: S1 Fig — Huh7 cells that had been pretreated with DMSO or actinomycin D for 1 hour were infected with HCV (MOI = 1) in the presence of DMSO or actinomycin D. After 2 hours of infection, the HCV inoculum was removed and cells were further incubated in fresh media for 8 hours. For the qRT-PCR analysis of HCV RNA, 100 ng total RNA was used. *, p<0.05. (TIFF) [file ppat.1004937.s001.tiff]

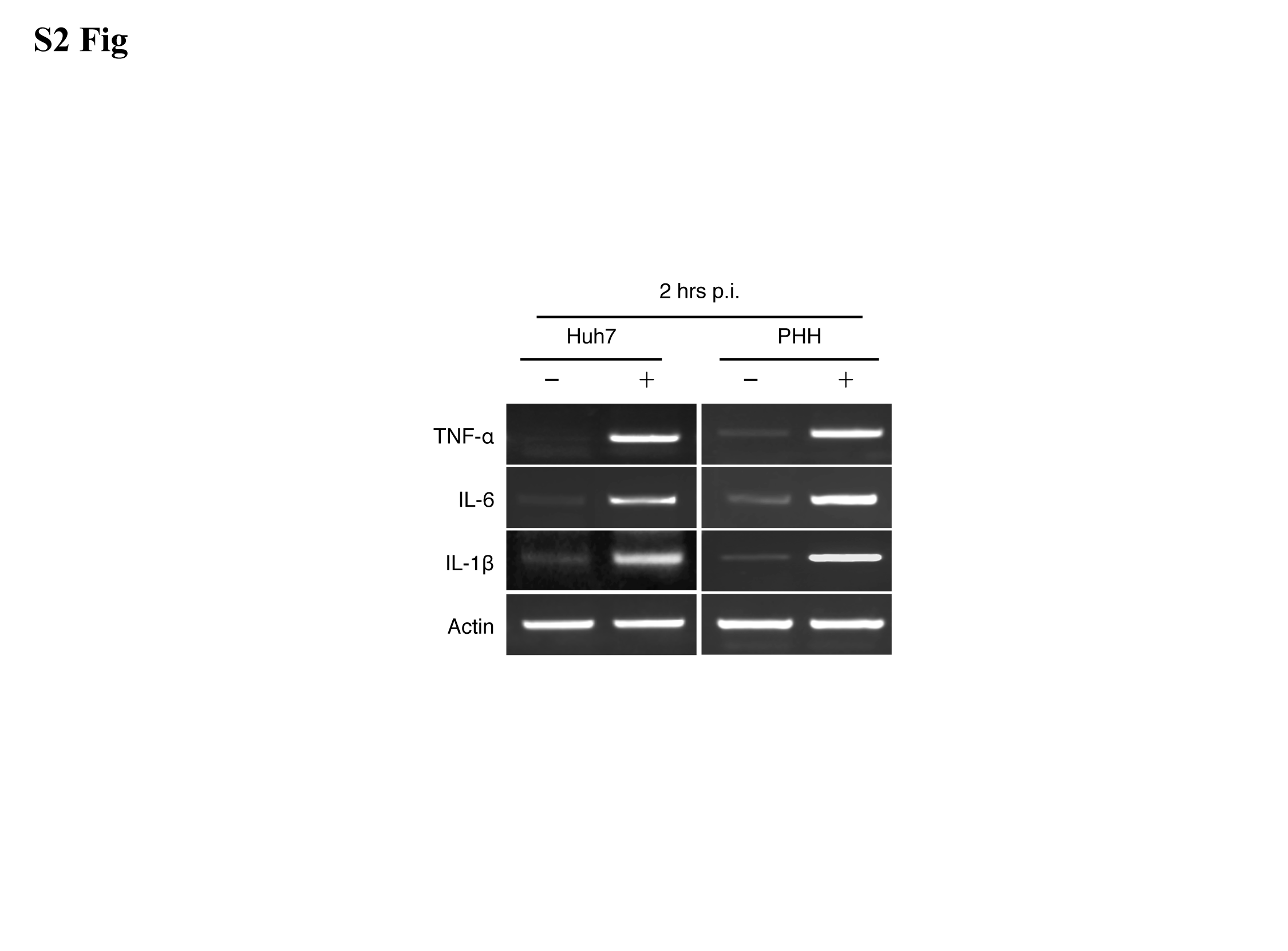

Supplement: S2 Fig — Huh7 cells or PHH were infected with HCV (MOI = 1) for 2 hours. Total cellular RNA was then isolated and analyzed by semi-quantitative RT-PCR for TNF-α, IL-6 and IL-1β RNAs. (TIFF) [file ppat.1004937.s002.tiff]

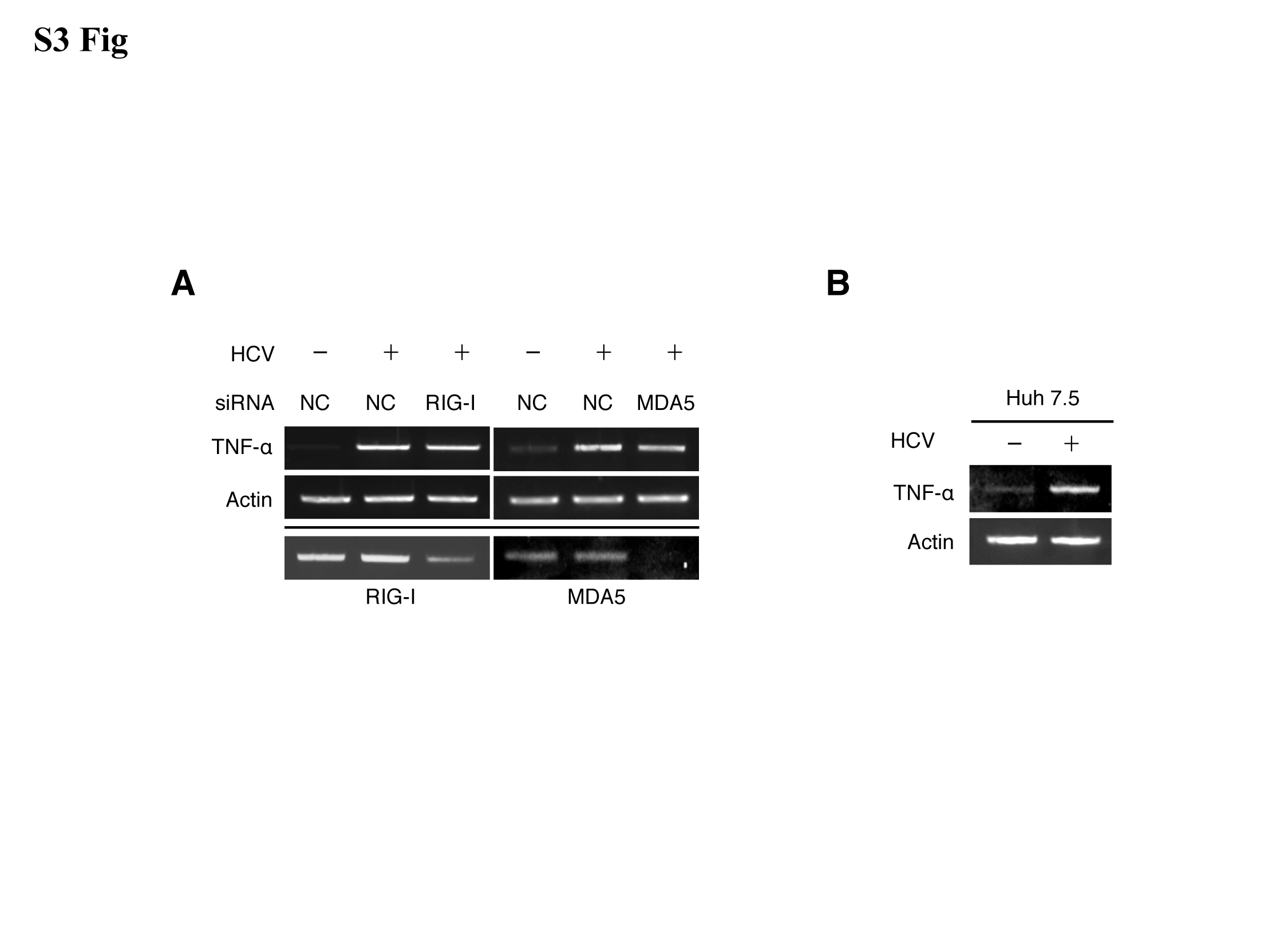

Supplement: S3 Fig — (A) Huh7 cells were transfected with the negative control (NC) siRNA or the siRNA directed against RIG-I or MDA5. At 48 hours post transfection, cells were infected with HCV (MOI = 1) for 2 hours. An equal amount of cellular RNA was subjected to RT-PCR for analysis of TNF-α induction and the knockdown efficiency of RIG-I and MDA5. (B) Huh7.5 cells were infected with HCV (MOI = 1) for 2 hours. Cells were then lysed for the RT-PCR analysis of TNF-α induction by HCV. (TIFF) [file ppat.1004937.s003.tiff]

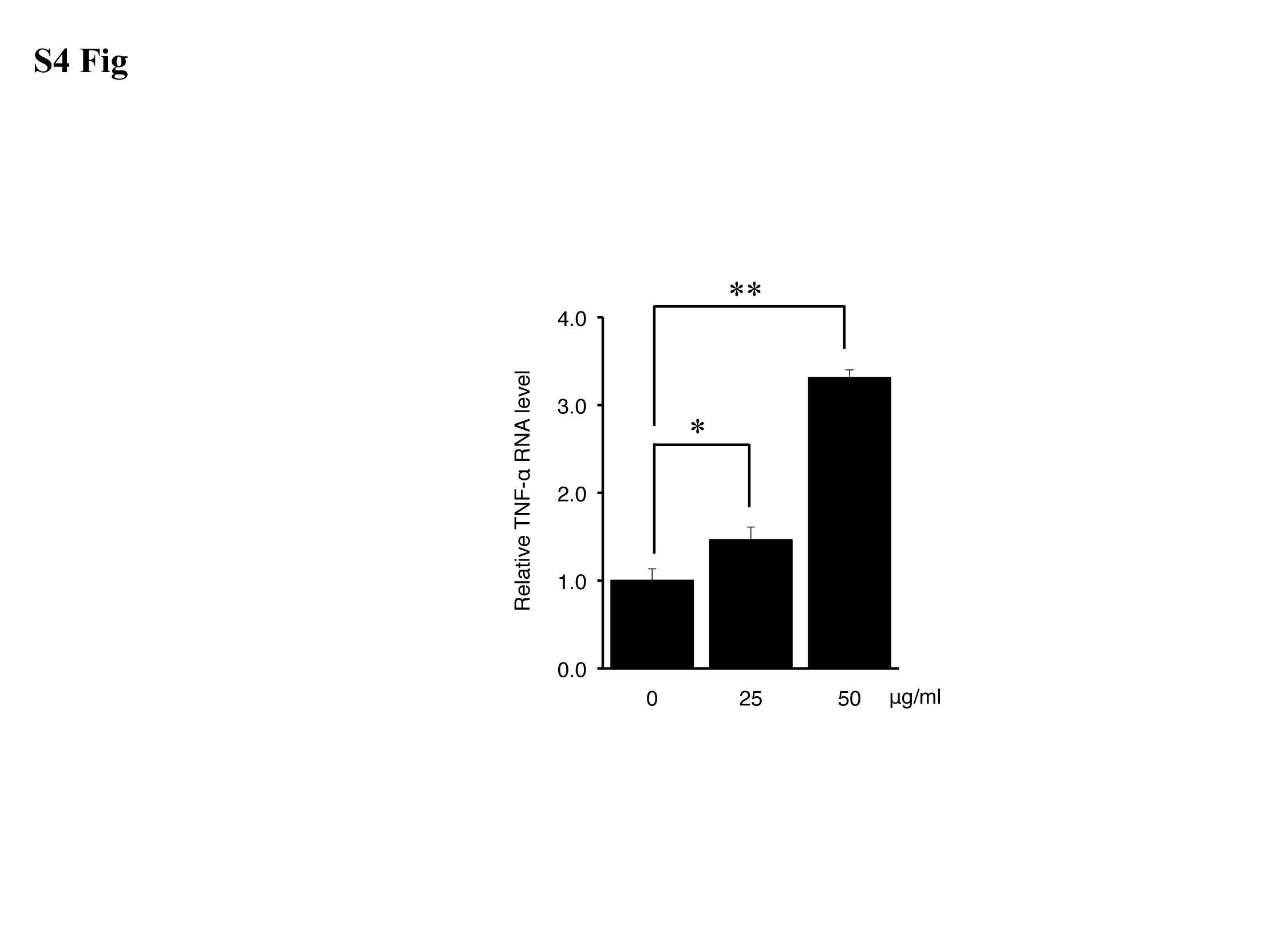

Supplement: S4 Fig — Huh7 cells were treated with 0, 25 or 50 μg/ml imidazoquinoline resiquimod (i.e., R848, the TLR7/8 agonist) for 24 hours. Total cellular RNA was analyzed for TNF-α RNA by qRT-PCR using GAPDH RNA as the control. * and **, p<0.05. (TIFF) [file ppat.1004937.s004.tiff]

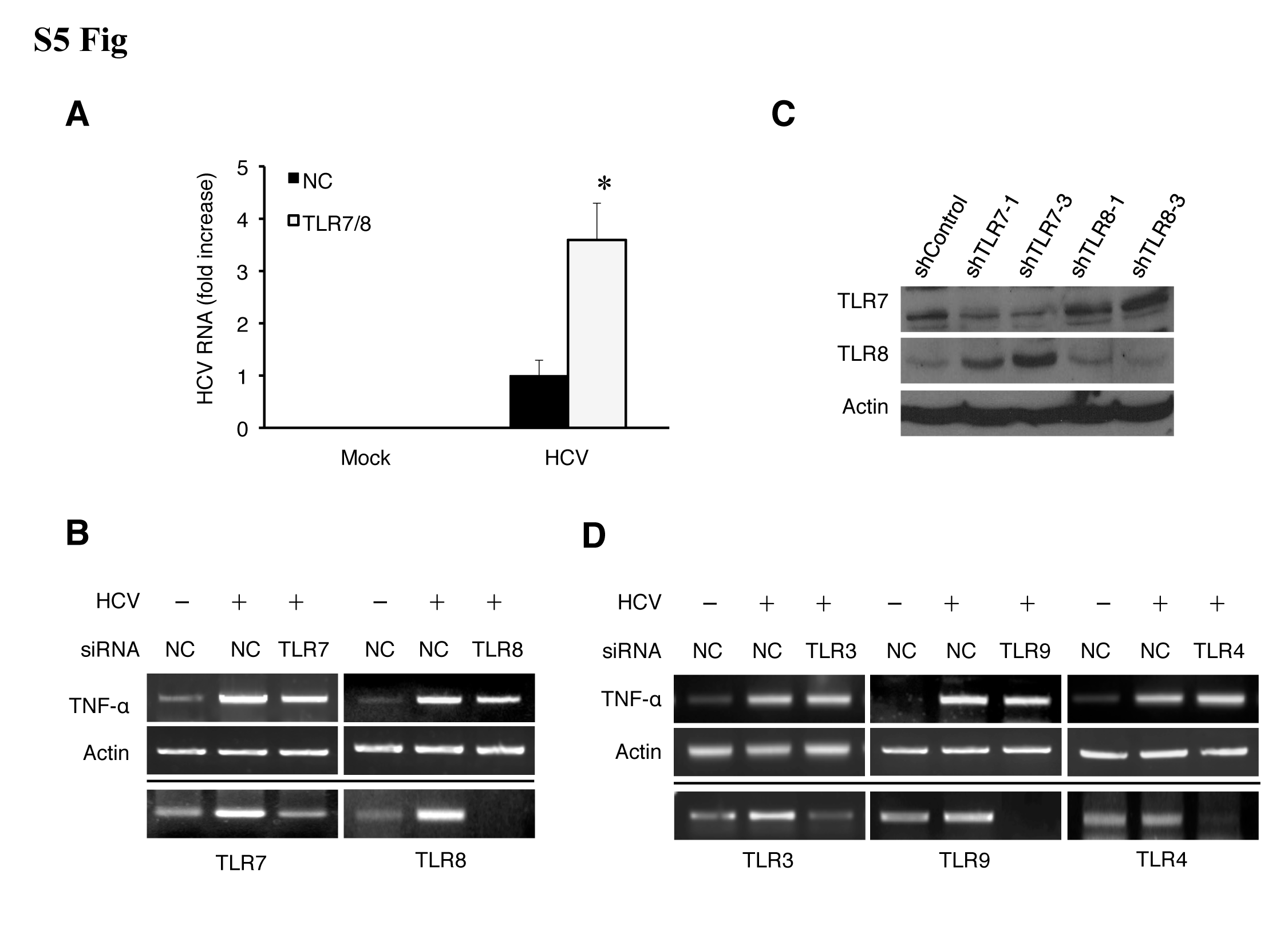

Supplement: S5 Fig — (A) Huh7 cells were transfected with NC siRNA or siRNAs targeting TLR7 and TLR8 for 6 hours, after which the siRNA complex was removed. At 48 hours post transfection, cells were mock-infected (-) or HCV-infected (+) (MOI = 1) for 24 hours. Total cellular RNA was then isolated for qRT-PCR analysis of HCV RNA. *, p<0.05. (B) Huh 7 cells were transfected with the control siRNA or siRNA targeting TLR7 or TLR8. After 48 hours, cells were infected with HCV (MOI = 1). Cells were lysed at 2 hours post-infection for RT-PCR analysis of various RNAs. (C) 293T cells were co-transfected with the pLKO.TRC plasmid (Addgene) that expressed TLR7 or TLR8 shRNA and the packaging vector (Addgene) for the production of recombinant lentiviral particles, which were harvested 60 hours post-transfection. Huh7 cells were then infected with these lentiviral particles and selected with puromycin (2μg/mL). Protein lysates were extracted from cell colonies 10 days post-selection and immunoblotted with antibodies against TLR7 and TLR8. shTLR7-1 and shTLR7-3 were two different shRNAs that targeted TLR7. Similarly, shTLR8-1 and shTLR8-3 were two different shRNAs that targeted TLR8. (D) The experiments were conducted using the same procedures mentioned above in (B), with the exception that TLR3, TLR4 and TLR9 siRNAs were used. (TIFF) [file ppat.1004937.s005.tiff]

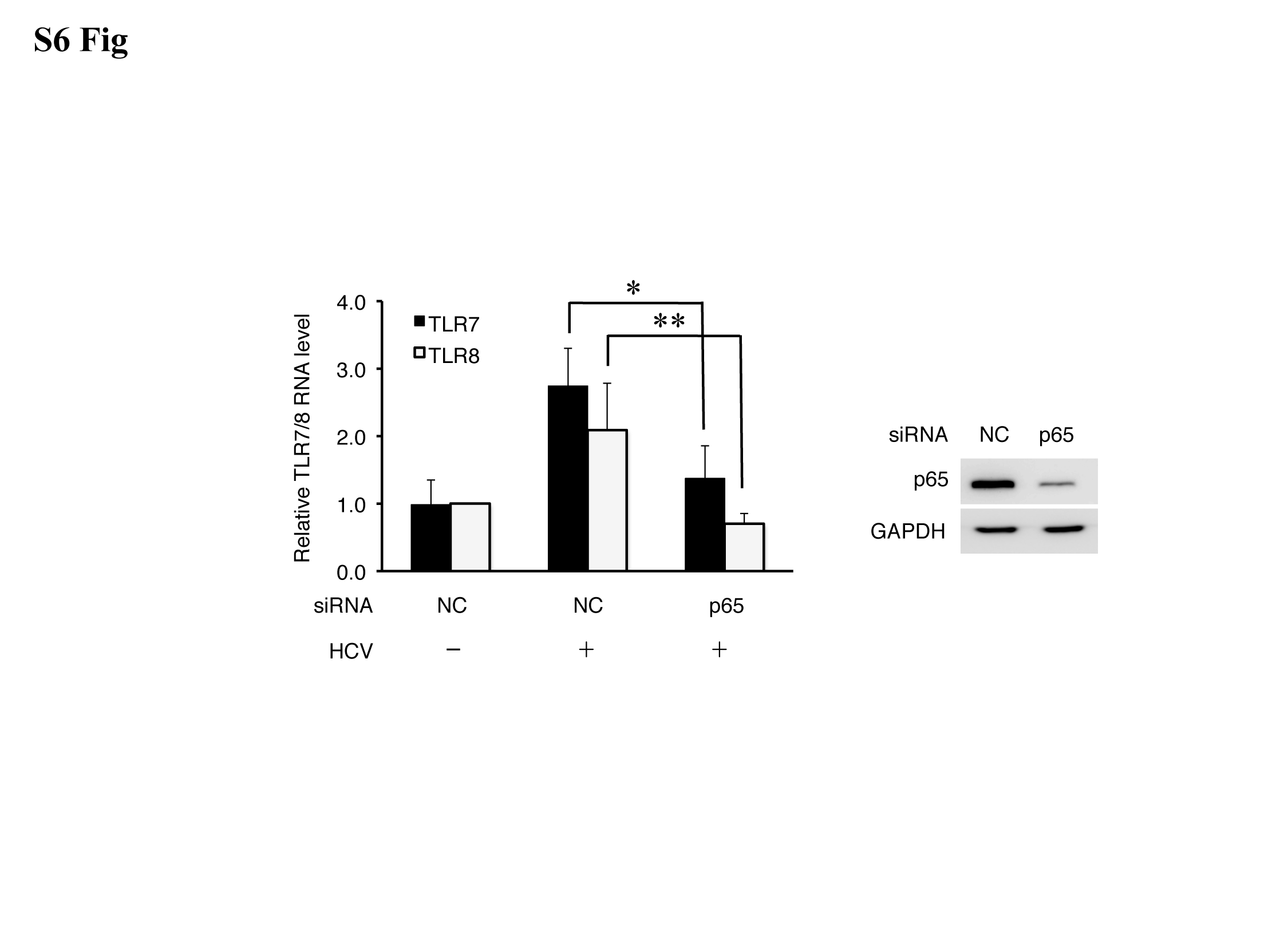

Supplement: S6 Fig — Huh 7 cells were transfected with the control siRNA or siRNA targeting p65 NFκB. At 48 hours after transfection, cells were infected with HCV (MOI = 1) for 2 hours. Total RNA was analyzed for TLR7 and TLR8 RNA expression using qRT-PCR. RNAs. The immunoblot of p65 was shown to the right for monitoring its knockdown efficiency. * and **, p<0.05. (TIFF) [file ppat.1004937.s006.tiff]

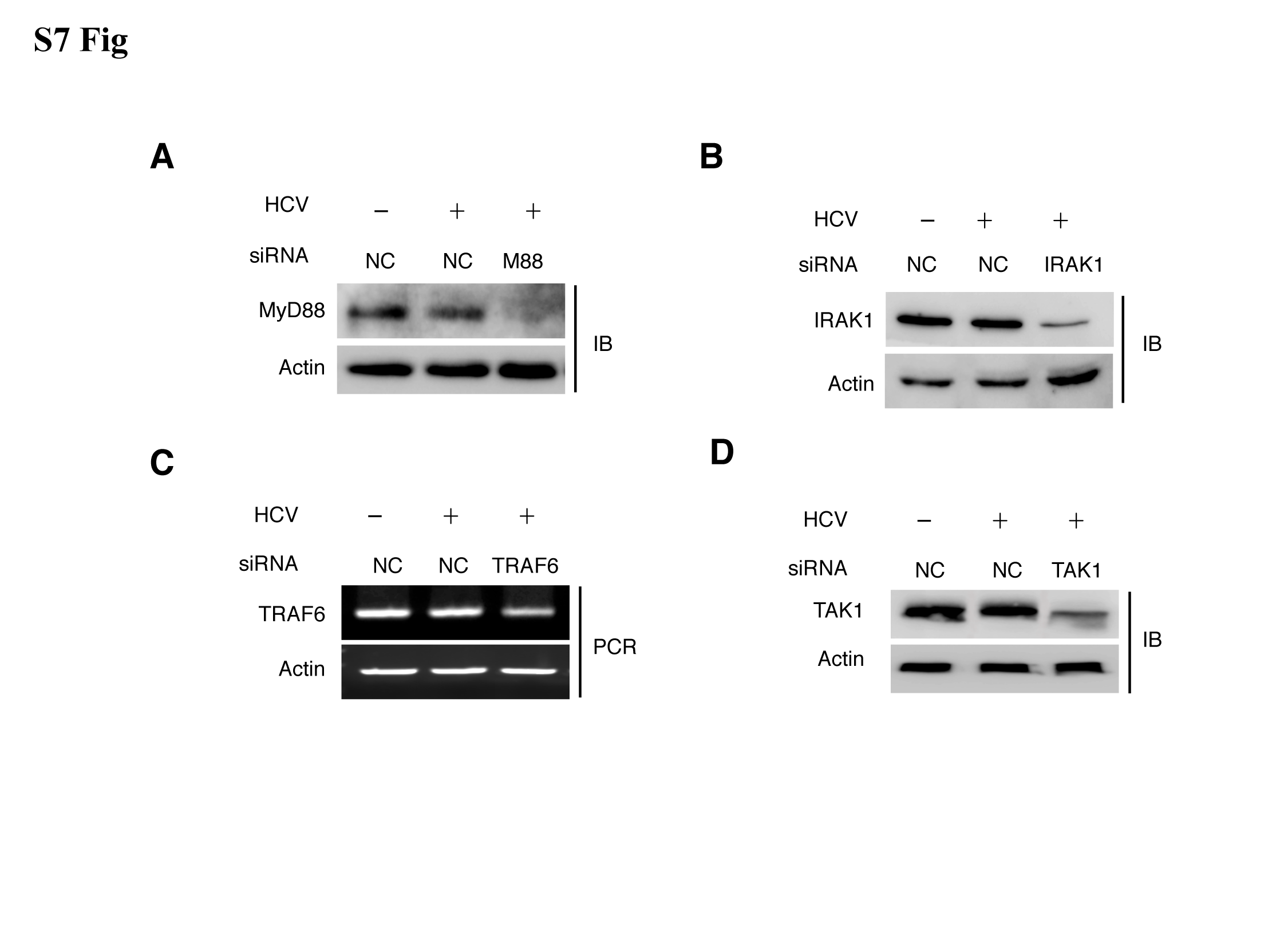

Supplement: S7 Fig — Huh7 cells were transfected with the negative control (NC) siRNA or siRNA targeting (A) MyD88, (B) IRAK1, (C) TRAF6, or (D) TAK1 two days prior to HCV infection. The transfected cells were then mock-infected or infected with HCV for 2 hours before being lysed for analysis. The knockdown efficiency of MyD88, IRAK1, and TAK1 was analyzed by immunoblotting (A, B, and D) and that of TRAF6 was analyzed by RT-PCR (C). (TIFF) [file ppat.1004937.s007.tiff]

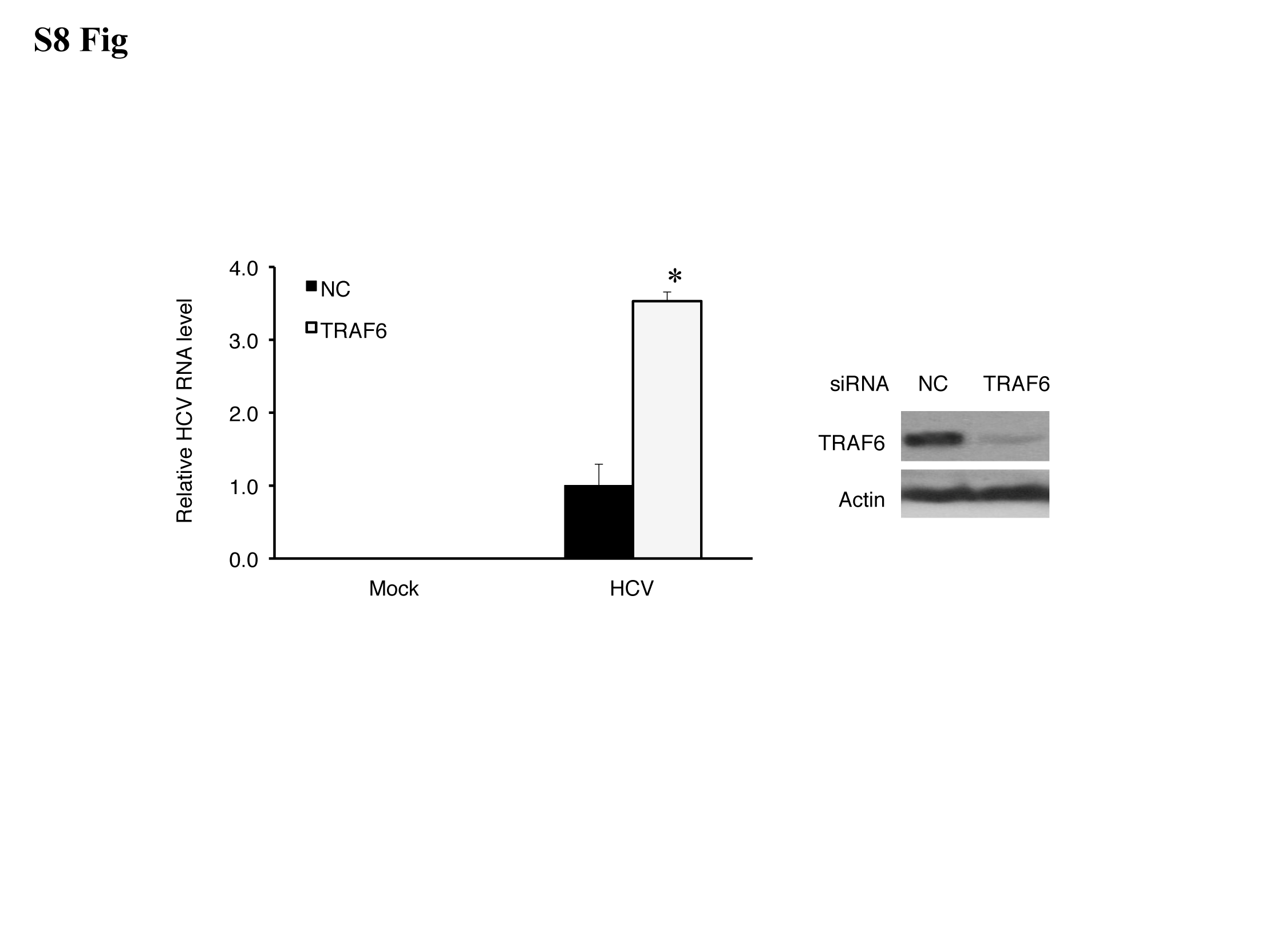

Supplement: S8 Fig — Huh7 cells were transfected with NC siRNA or siRNAs targeting TRAF6. Forty-eight hours post-transfection, cells were mock-infected (-) or HCV-infected (+) (MOI = 1) for 24 hours. Total cellular RNA was then isolated for qRT-PCR analysis of TNF-α RNA. *, p<0.05. (TIFF) [file ppat.1004937.s008.tiff]

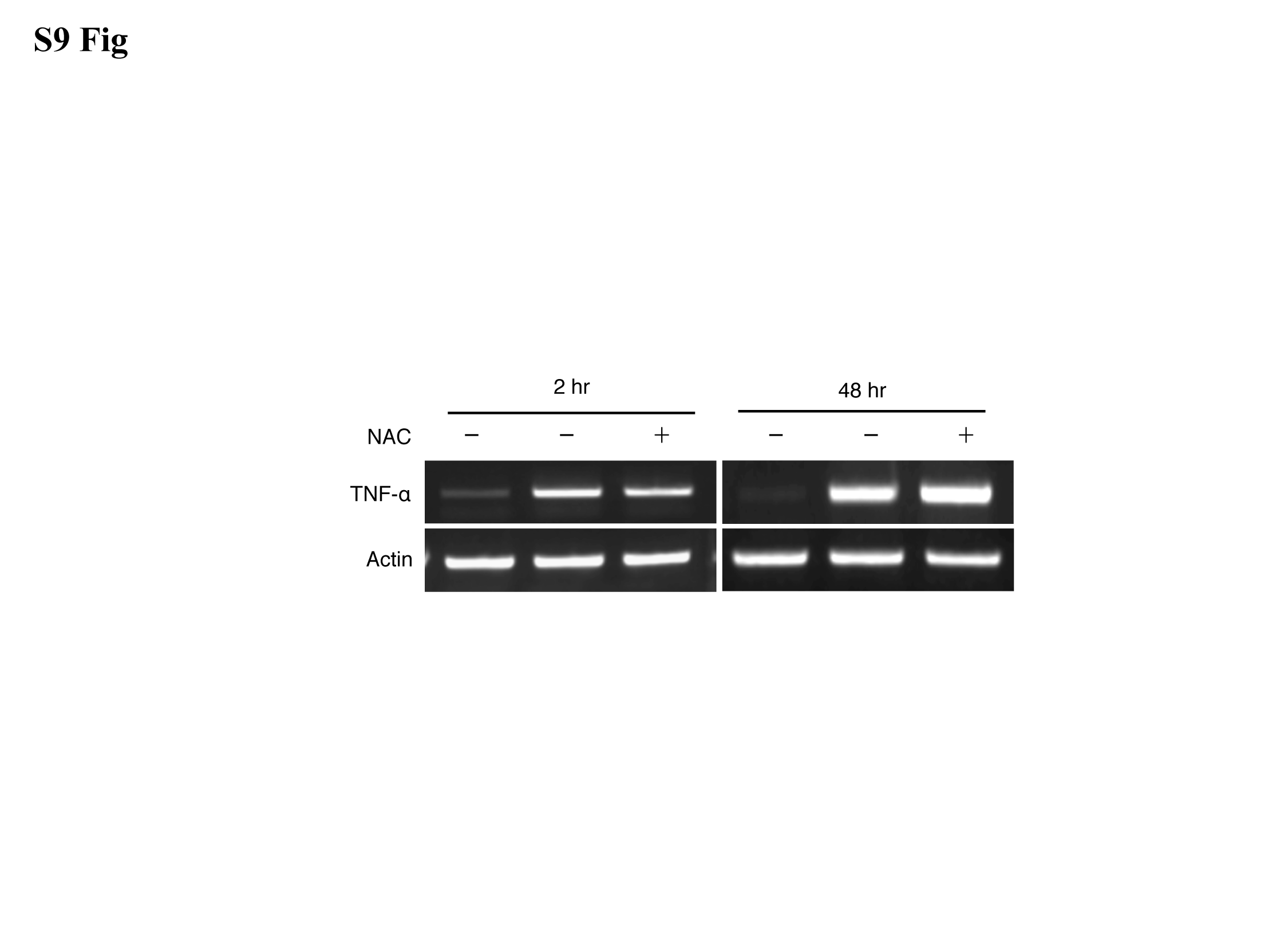

Supplement: S9 Fig — Huh7 cells were pretreated with NAC for one hour prior to infection with HCV (MOI = 1) and for another two hours after HCV infection, or with NAC during the final six hours of the 48-hour infection. Total RNA was isolated for RT-PCR analysis of TNF-α RNA. (TIFF) [file ppat.1004937.s009.tiff]

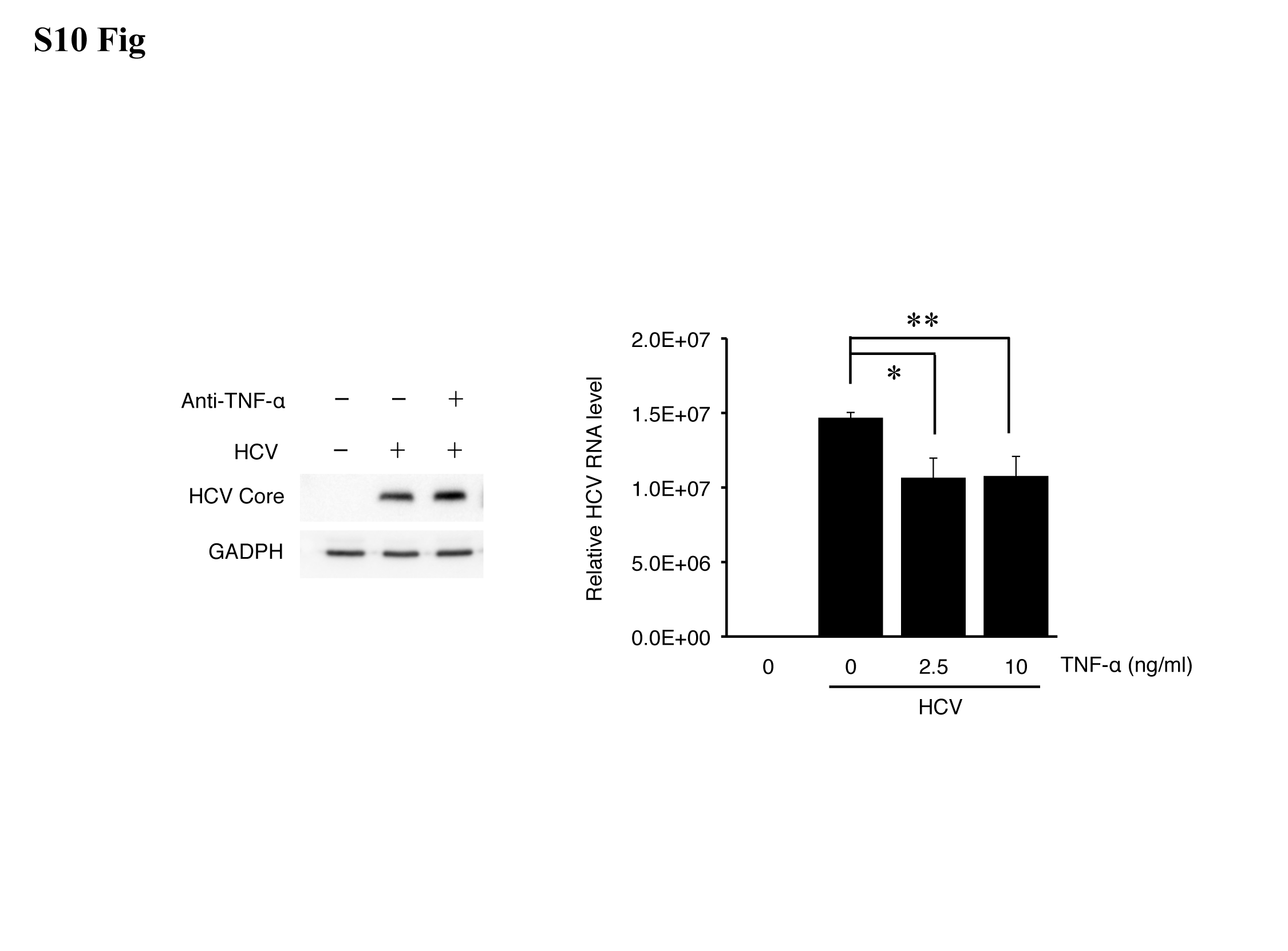

Supplement: S10 Fig — Huh7 cells were pretreated with recombinant human TNF-α (R&D systems) for 4 hours prior to and during infection with HCV (MOI = 1). Total cellular RNA was isolated at 20 hours post-infection for qRT-PCR analysis of HCV RNA. * and **, p<0.05. (TIFF) [file ppat.1004937.s010.tiff]

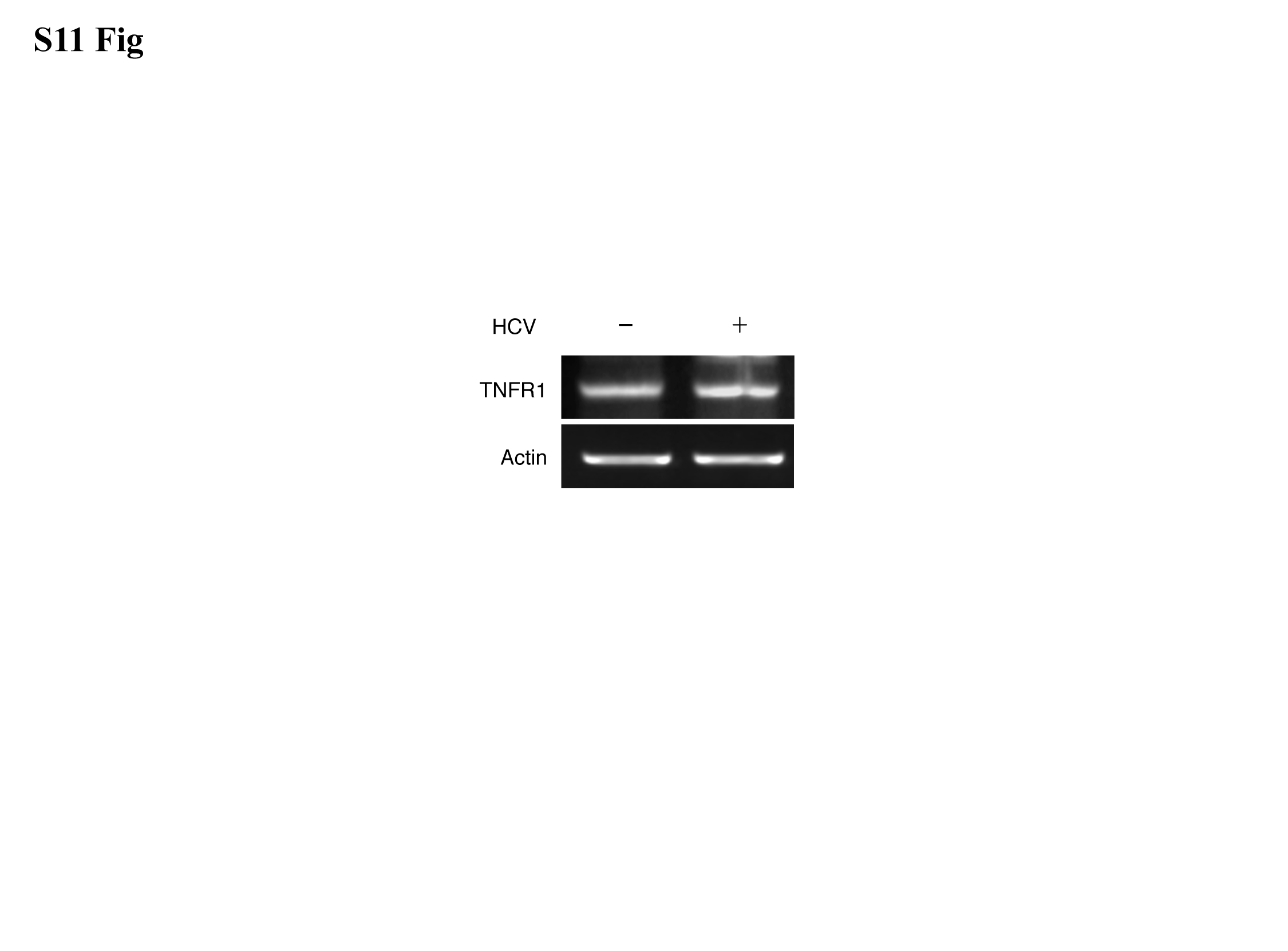

Supplement: S11 Fig — Huh7 cells with or without HCV infection for 48 hours were lysed for the isolation of total RNA, which was then analyzed for TNFR1 RNA by semi-quantitative RT-PCR. (TIFF) [file ppat.1004937.s011.tiff]

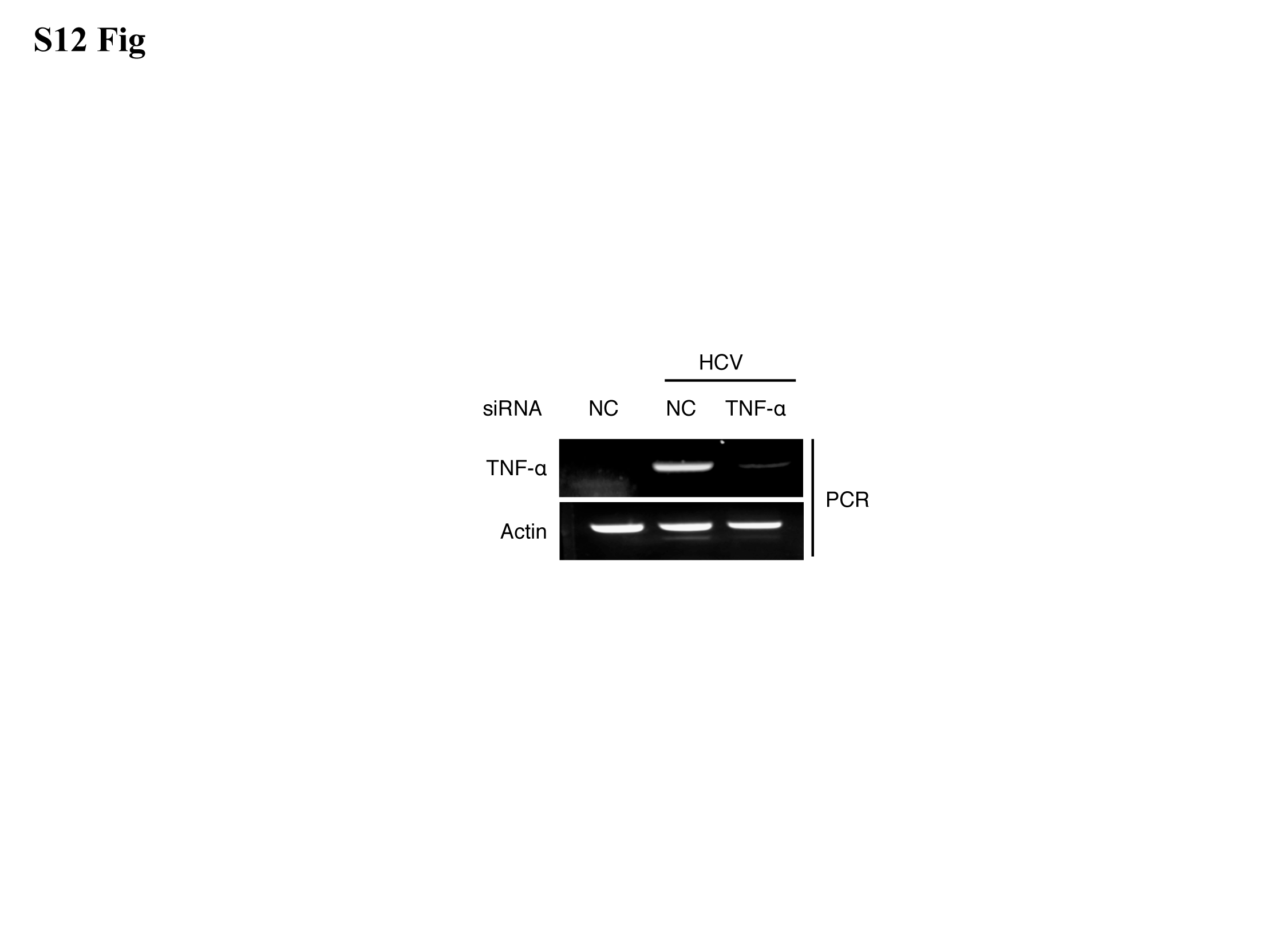

Supplement: S12 Fig — The experiments were conducted as described in the legend of Fig 6A and 6B. The knockdown efficiency of TNF-α was analyzed by semi-quantitative RT-PCR. (TIFF) [file ppat.1004937.s012.tiff]

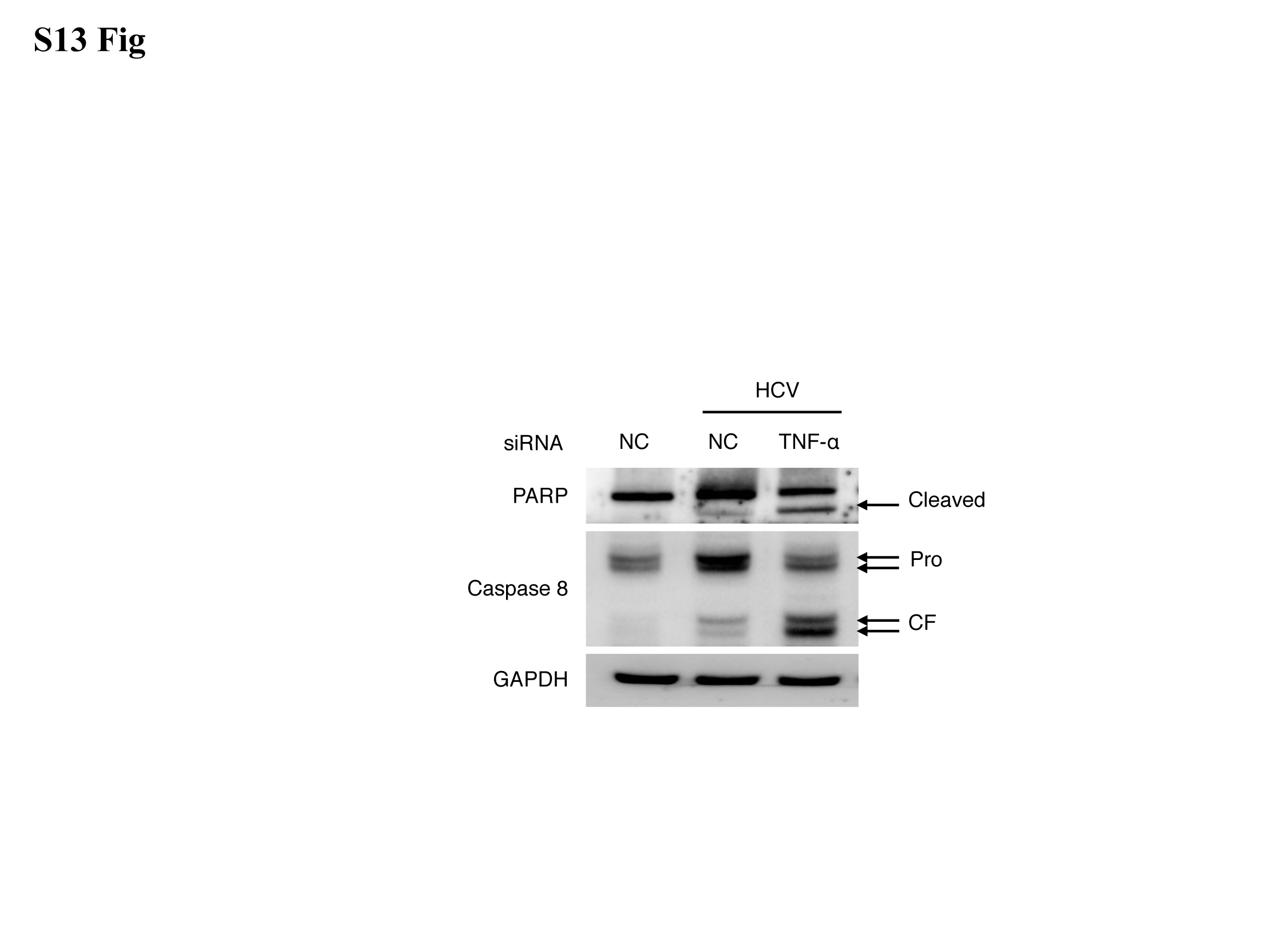

Supplement: S13 Fig — Huh7 cells were transfected with negative control (NC) siRNA or TNF-α siRNA. At 48 hours post transfection, cells were mock-infected or HCV-infected (MOI = 0.25) for 48 hours. Cells were then lysed for immunoblot analysis of poly (ADP-ribose) polymerase (PARP) and caspase 8. GAPDH was used as an internal loading control. The locations of cleaved PARP, procaspase 8 (Pro) and cleaved fragments (CF) of caspase 8 are indicated. Note that the knockdown of TNF-α enhanced apoptosis, as evidenced by the enhanced cleavage of PARP and procaspase 8, which are markers of apoptosis. (TIFF) [file ppat.1004937.s013.tiff]

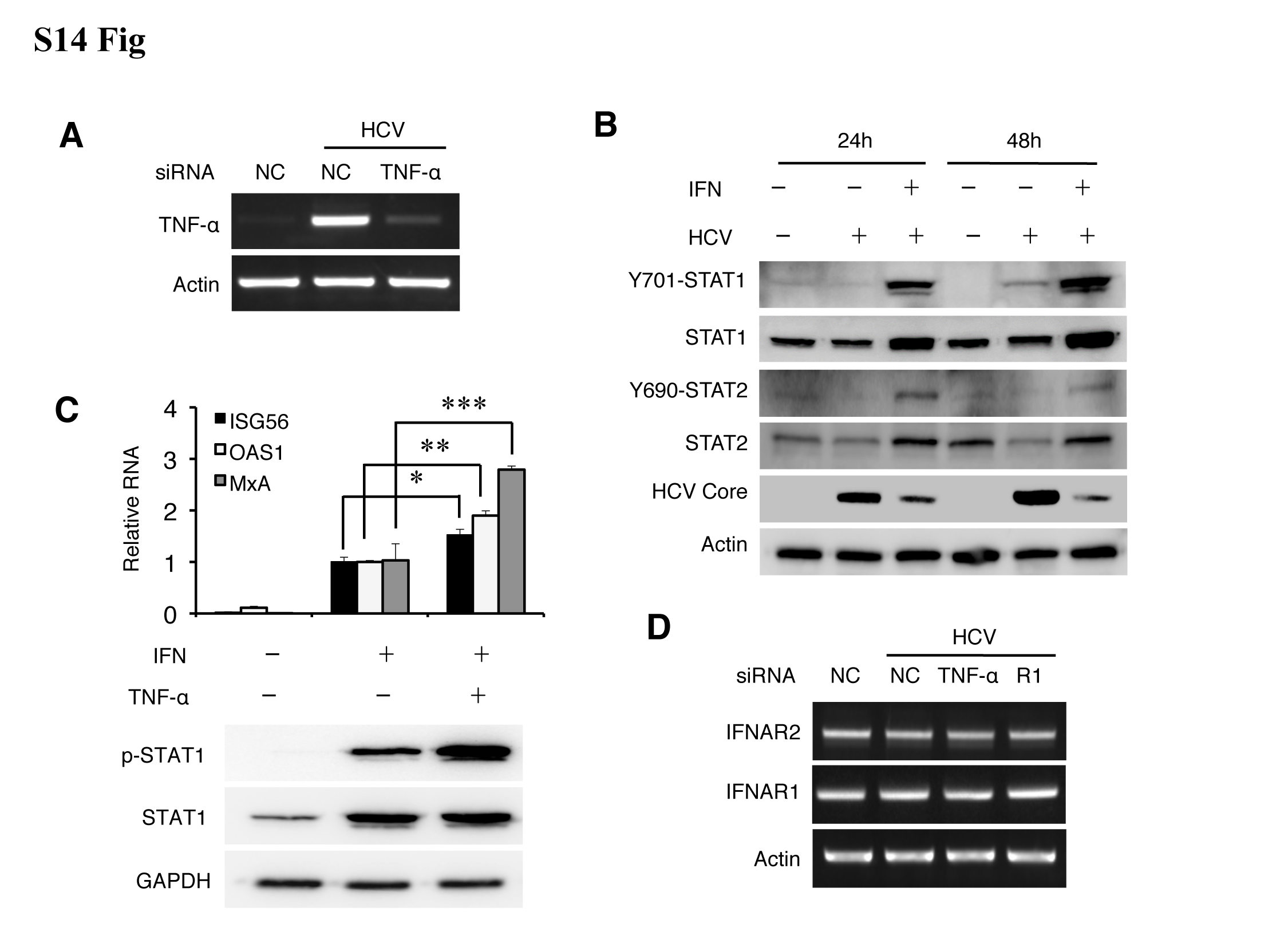

Supplement: S14 Fig — (A) Analysis of the knockdown efficiency of TNF-α, which was analyzed by semi-quantitative RT-PCR. The actin RNA was also analyzed to serve as the internal control. This study is to support Fig 7A. (B) Huh7 cells were infected with HCV for either 4 hours or 30 hours and then further incubated with or without IFN-α (1000 units) for 18 hours. Cells were then lysed at either 24 hours or 48 hours post-infection for immunoblot analysis of the phosphorylation status of STAT1 and STAT2. Total STAT1 and STAT2 and the HCV core expression were also analyzed. Actin served as the loading control. (C) Huh7 cells were incubated with IFN-α in absence or presence of TNF-α for 24 hours. STAT1 phosphorylation was analyzed by western blot, and relative ISG expression levels were analyzed by qRT-PCR. *, p = 0.01, **, p = 8.5E-05, ***, p = 0.005. (D) Huh7 cells were transfected with NC-, TNF-α-, or TNFR1-siRNA. After 48 hours, the siRNA transfection was repeated. Cells were infected with HCV at 6 hours after the second siRNA transfection. Total cellular RNA was isolated at 48 hours post-infection and analyzed by semi-quantitative RT-PCR for IFNAR1 and IFNAR2 RNAs. The actin RNA was also analyzed to serve as the control. (TIFF) [file ppat.1004937.s014.tiff]

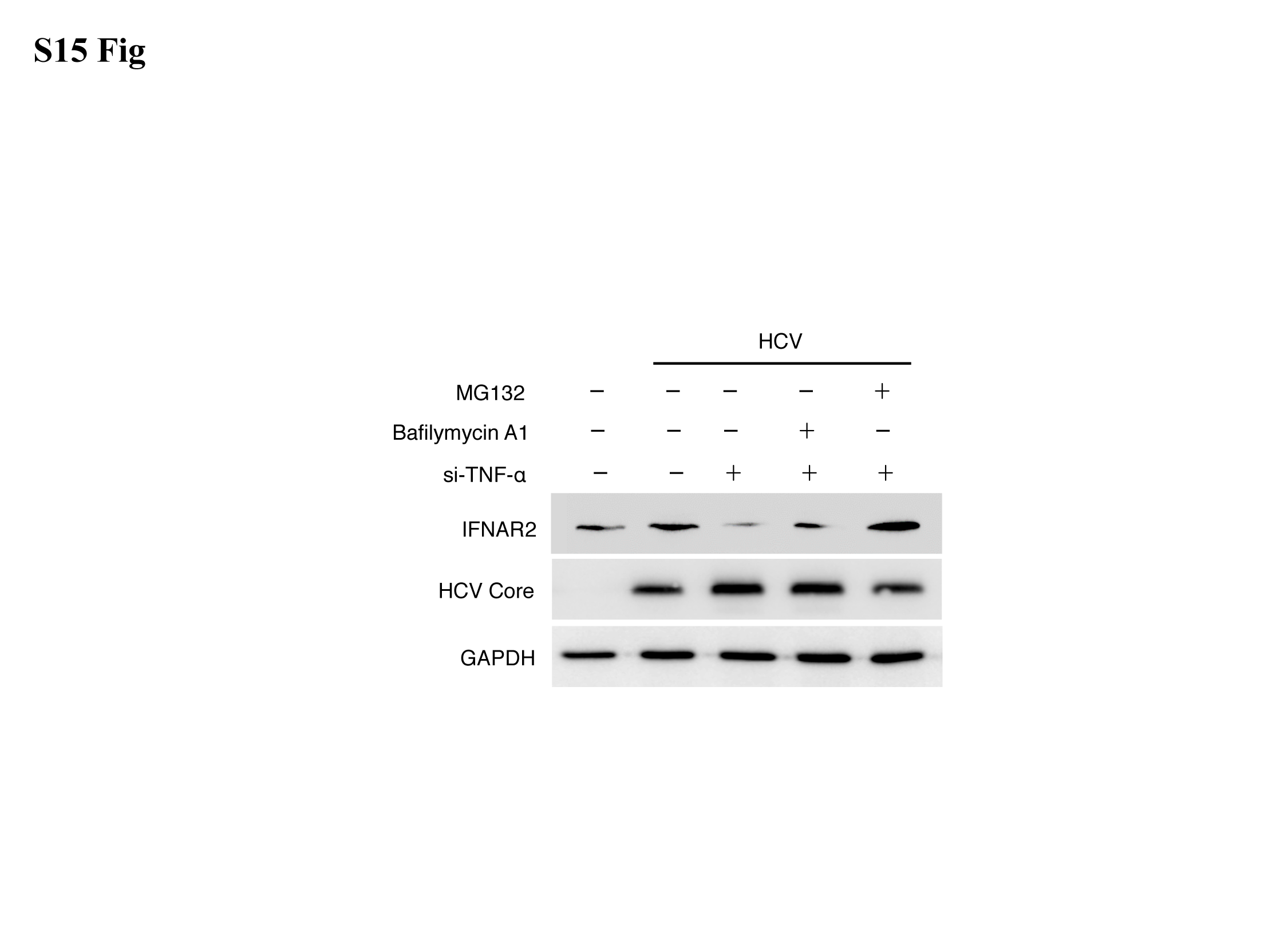

Supplement: S15 Fig — Huh7 cells were transfected with the negative control siRNA or TNF-α siRNA. After 48 hours, cells were infected with HCV (MOI = 0.25) for 24 hours and then treated with DMSO, 200 nM Bafilomycin A1, or 10 μM MG132 for additional 16 hours. Cells were then lysed for immunoblot analysis of IFNAR2 and the HCV core protein. GAPDH served as the loading control. (TIFF) [file ppat.1004937.s015.tiff]

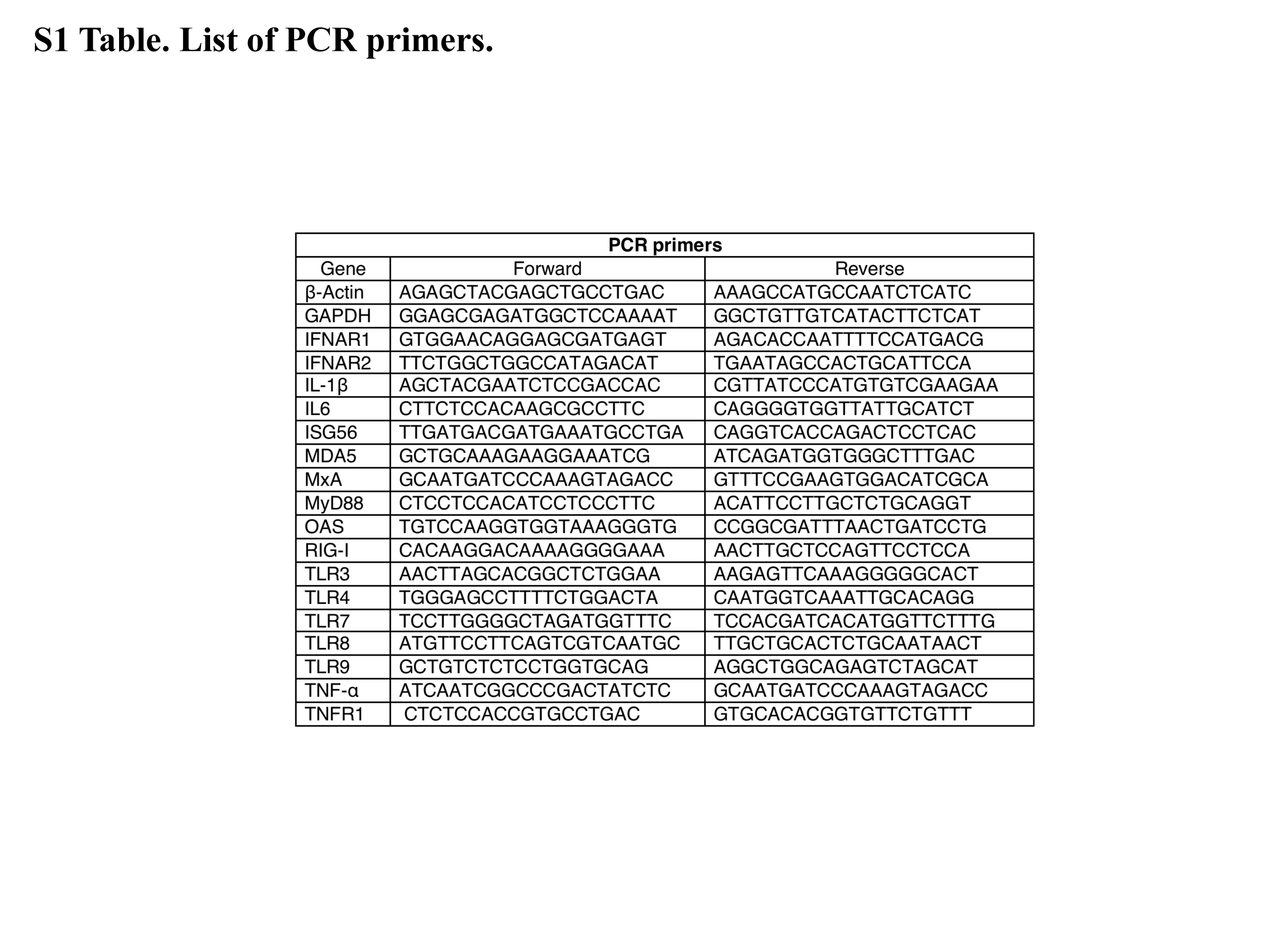

Supplement: S1 Table — (TIFF) [file ppat.1004937.s016.tiff]
